# A *Culex quinquefasciatus* strain resistant to the binary toxin from *Lysinibacillus sphaericus* displays altered enzyme activities and energy reserves

**DOI:** 10.1186/s13071-023-05893-z

**Published:** 2023-08-09

**Authors:** Heverly Suzany G. Menezes, Samara G. Costa-Latgé, Fernando A. Genta, Thiago H. Napoleão, Patrícia M. G. Paiva, Tatiany P. Romão, Maria Helena N. L. Silva-Filha

**Affiliations:** 1 Department of Entomology, Instituto Aggeu Magalhães-FIOCRUZ, Av. Moraes Rego s/n, Recife, PE 50740-465 Brazil; 2 Laboratory of Insect Biochemistry and Physiology, Instituto Oswaldo Cruz-FIOCRUZ, Rio de Janeiro, RJ 21045-900 Brazil; 3 National Institute for Molecular Entomology, Rio de Janeiro, RJ 21941-902 Brazil; 4 Department of Biochemistry, Universidade Federal de Pernambuco, Recife, PE 50670-901 Brazil

**Keywords:** Mosquito control, Biolarvicides, Transcriptome, Lipids, Glycogen, Fitness

## Abstract

**Background:**

The resistance of a *Culex quinquefasciatus* strain to the binary (Bin) larvicidal toxin from *Lysinibacillus sphaericus* is due to the lack of expression of the toxin’s receptors, the membrane-bound Cqm1 α-glucosidases. A previous transcriptomic profile of the resistant larvae showed differentially expressed genes coding Cqm1, lipases, proteases and other genes involved in lipid and carbohydrate metabolism. This study aimed to investigate the metabolic features of Bin-resistant individuals by comparing the activity of some enzymes, energy reserves, fertility and fecundity to a susceptible strain.

**Methods:**

The activity of specific enzymes was recorded in midgut samples from resistant and susceptible larvae. The amount of lipids and reducing sugars was determined for larvae and adults from both strains. Additionally, the fecundity and fertility parameters of these strains under control and stress conditions were examined.

**Results:**

Enzyme assays showed that the esterase activities in the midgut of resistant larvae were significantly lower than susceptible ones using acetyl-, butyryl- and heptanoyl-methylumbelliferyl esthers as substrates. The α-glucosidase activity was also reduced in resistant larvae using sucrose and a synthetic substrate. No difference in protease activities as trypsins, chymotrypsins and aminopeptidases was detected between resistant and susceptible larvae. In larval and adult stages, the resistant strain showed an altered profile of energy reserves characterized by significantly reduced levels of lipids and a greater amount of reducing sugars. The fertility and fecundity of females were similar for both strains, indicating that those changes in energy reserves did not affect these reproductive parameters.

**Conclusions:**

Our dataset showed that Bin-resistant insects display differential metabolic features co-selected with the phenotype of resistance that can potentially have effects on mosquito fitness, in particular, due to the reduced lipid accumulation.

**Graphical Abstract:**

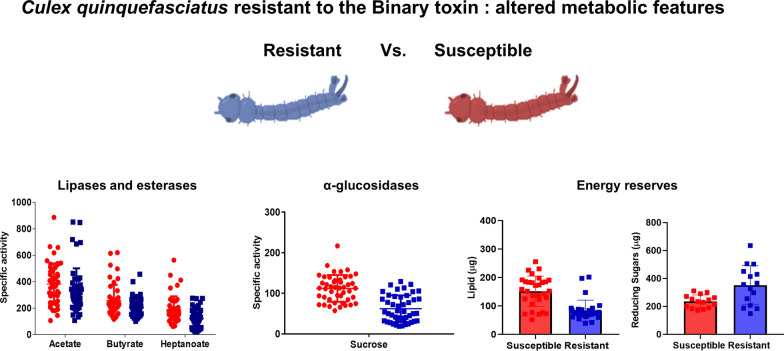

**Supplementary Information:**

The online version contains supplementary material available at 10.1186/s13071-023-05893-z.

## Background

Microbial larvicides based on *Lysinibacillus sphaericus* have been successfully used to control *Culex* and *Anopheles* in several countries [1–4]. These larvicides are based on the Binary (Bin) protoxin, a heterodimeric protein found in crystals produced during bacterial sporulation [5]. The activity of the Bin protoxin on *Culex quinquefasciatus* depends on the ingestion of crystals by larvae and processing of the protoxin into an active toxin. Then, the Bin toxin specifically binds to the Cqm1 receptors, which are α-glucosidases attached to the epithelial midgut cells by a glycosylphosphatidylinositol (GPI) anchor [3, 6]. The resistance to the Bin toxin is the main difficulty related to the use of *L. sphaericus*-based larvicides, and it has been reported in *Cx. quinquefasciatus* laboratory-selected strains and field-treated populations [3, 7, 8]. For this reason, the utilization of *L. sphaericus* Bin crystals should be associated with other compounds, particularly with Bti [4, 9–11]. This is because, together, the crystals of both bacteria can display a synergistic action to prevent the onset of resistance [12–14]. In most cases, the resistance to the Bin toxin is provoked by the absence of the midgut Cqm1 receptors [15, 16] because of mutations found in alleles of the *cqm1* gene that prevent the expression of membrane-bound receptors, making these larvae refractory to the Bin toxin [17–21].

Among the *Cx. quinquefasciatus*-resistant cases already reported [3], the RIAB59 is a laboratory strain selected with the *L. sphaericus* IAB59 [22] that exhibits a high resistance ratio to the Bin toxin (RR > 5000-fold). The RIAB59 individuals carry the *cqm1*_*REC*_ allele in homozygosity, which causes the lack of the GPI-anchored Cqm1 α-glucosidases in the midgut epithelium [23]. The RIAB59 strain also displays a moderate resistance level (RR ~ 15-fold) to the Cry48Aa/Cry49Aa toxin. This is a second binary toxin produced by the IAB59 strain which binds to other receptors than the Cqm1 [24–26]. Most investigations on *L. sphaericus* resistance have concentrated efforts to characterize the mechanisms and genes that confer the Bin-resistance itself [3], while much less is known about other features that could be related to this phenotype. This is particularly important for Bin-resistant strains, such as RIAB59, that have been stably maintained for several generations without a critical impact on their biological traits [22, 23, 27, 28].

A transcriptomic profile of larvae from the RIAB59 strain provided a broader molecular view of the Bin-resistance as it revealed a remarkable set of significantly differential expressed genes (DEGs) compared to the susceptible strain [29]. The *cqm1* transcript, which is a marker of Bin resistance, was detected among the most downregulated genes, which was consistent with the respective phenotype and the homozygous *cqm1*_*REC*_ genotype of the RIAB59 individuals [17]. Most importantly, a significantly altered expression profile of other genes, several of them involved in lipid and carbohydrate metabolism, was found in resistant individuals compared to the susceptible counterparts. The lipid reserve is one of the most important used by insects because of the high energy consumption for metamorphosis [30], embryo development and flight [31, 32]. Carbohydrates are mobilized mainly from glycogen reserves in the fat body. The amount of glycogen is lower than the fat and displays variations according to motor and feeding activity. After metamorphosis, glycogen is dramatically reduced and can be nearly depleted, and adults recover glycogen stores only after feeding on sugar sources [31]. Therefore, in this study, we hypothesized that RIAB59-resistant individuals display an altered activity of some enzymes, as suggested by the differentiated transcriptomic profile, potentially affecting their nutritional reserves. To determine whether the high resistance of this strain to the Bin larvicidal toxin was related to differential features, we compared the resistant and susceptible *Cx. quinquefasciatus* strains in terms of the catalytic activity of esterases, lipases, α-glucosidases and proteases, storage of lipids and reducing sugars and evaluation of fertility and fecundity.

## Methods

### *Culex quinquefasciatus* strains

All experiments were conducted using a susceptible (CqSLab, here denominated S) and the resistant (RIAB59, here denominated R) strains maintained at the insectary of Instituto Aggeu Magalhães (IAM-FIOCRUZ). The S strain was established with eggs collected in Recife city (Brazil), and it has been maintained for > 10 years. The R strain, derived from the same area, has been selected with *L. sphaericus* IAB59 [22] and achieved a high level of resistance to the Bin toxin and a moderate resistance level to the Cry49Aa/Cry48Aa toxin [26], as described in the introduction. The high Bin resistance was achieved after selection during 46 generations [33]; after this, the strain has been exposed to *L. sphaericus* IAB59 at every five generations to periodically confirm the status of in vivo resistance. A summary of the outstanding DEGs found in the transcriptomic profile of the midgut of R larvae is shown in Additional file 1: Table S1. This analysis was carried out using R individuals from the generations F_250_ and F_259_. Both strains were maintained under controlled temperature (26 ± 1 °C), relative humidity (70%) and photoperiod (14 h light:10 h dark). Adults were fed with sucrose solution 10% (*w*/*v*), and females were also fed with defibrinated rabbit blood provided by ICTB-Fiocruz (Rio de Janeiro, RJ, Brazil). Larvae were reared in tap water and fed cat food (Whiskas^®^, Ribeirão Preto, SP, Brazil). For this, larvae from both strains investigated in this study were reared under the following optimal conditions, unless when specified. First instar larvae from one raft (~ 250 eggs) were set in a plastic tray (21.5 cm weight × 29 cm large × 5.5 cm high) filled with tap water (1 l), and cat food was provided (0.8 g) during day 0 (0.1 g), day 4 (0.3 g) and day 8 (0.4 g). Adults from these larvae were collected and fed as described before.

### Midgut sample preparation for protein and enzyme measurements

Whole fourth instar larvae midguts, with their contents involved by the peritrophic membrane, were individually dissected in saline solution (NaCl 0.9%, *w*/*v*) under ice, homogenized in an appropriate buffer described in each section and centrifuged (15,000 ×*g*, 4 °C, 10 min). The supernatant was collected for the assays. For all enzyme classes evaluated, except for the proteases, a mix of protease inhibitors was used in the reaction buffers composed by the following components (Sigma-Aldrich, St. Louis, MO, USA) at the following final concentrations: 20 µM E-64 (n-trans-epoxysuccinyl-l-leucine4-guanidinobutylamide), 20 µM pepstatin A and 10 mM PMSF (phenylmethylsulfonyl fluoride). The protein concentration was determined using the Pierce™ BCA Protein Assay Kit [34], according to the manufacturer's instructions (Thermo Fisher Scientific, Waltham, MA, USA), using bovine serum albumin (BSA) for the standard curve. Enzymatic and protein quantification assays were performed using individual midguts tested in duplicate. Each assay was repeated at least three times with a minimum of 15 insects per biological replicate. One unit of enzymatic activity (U) is defined as the amount of enzyme which releases 1 µmol of product/min. Preliminary experiments were performed to determine the optimal conditions for performing the enzymatic assays.

### Lipase and esterase assays

The total activities of lipases (EC 3.1.1.34) and esterases (EC 3.1.1.1) were measured in whole midgut homogenate samples using continuous assays with fluorescence detection. Five different substrates (Sigma-Aldrich) were tested at the final concentrations indicated: 4-methylumbelliferyl acetate (10 µM), 4-methylumbelliferyl butyrate (10 µM), 4-methylumbelliferyl heptanoate (10 µM), 4-methylumbelliferyl palmitate (100 µM; BioChemika Fluka, St. Louis, MO, USA) and 4-methylumbelliferyl oleate (100 µM). Each midgut was homogenized in 100 µl of saline solution (0.9%) containing the mix of protease inhibitors. The reaction was composed by the midgut sample (20 µl for acetate, butyrate and heptanoate; 25 µl for oleate; 40 µl for palmitate) and each substrate in a 200-µl final reaction volume of 200 mM Tris–HCl buffer (pH 8.5) in 96-well plates. These were incubated at 30 °C for 20 min (acetate, butyrate and oleate) or for 60 min (heptanoate and palmitate). The fluorescence detection of 4-methylumbelliferone was continuously recorded at 360 nm of excitation and 449 nm of emission in the SpectraMax Gemini XPS^™^ 96-well microplate reader (Molecular Devices, San Jose, CA, USA). The amount of reaction product released was calculated using a standard curve of 4-methylumbelliferone (0.1–2 nmol) recorded under the same conditions as the test samples. A total number from 41 to 48 individual midgut samples was analyzed per strain.

### α-Glucosidase assays

The α-glucosidase activity (E.C. 3.2.1.20) was determined in whole midgut individual samples that were homogenized in 100 µl of citrate–phosphate buffer (200 mM, pH 6.5), containing 1% (*v*/*v*) Triton-X. The activity was measured using the synthetic substrate 4-methylumbelliferyl α-glucopyranoside (MUαGlu, Sigma-Aldrich) or natural substrate sucrose (Sigma-Aldrich). The reaction was composed by the midgut sample (50 µl), 0.4 mM MUαGlu in a 200 µl-final reaction volume of the citrate–phosphate buffer, which was incubated at 30 °C for 1 h. The 4-methylumbelliferone released was determined continuously at excitation of 355 nm and emission of 460 nm [35] in the SpectraMax Gemini XPS^™^ microplate reader. The amount of reaction product released was calculated from a standard curve of 4-methylumbelliferone (0.3–6 nmol) read under the same conditions as the test samples. For the assays using sucrose as substrate, the reaction was composed by the midgut sample (20 µl), 200 mM sucrose (Sigma-Aldrich) in a 50 µl-final reaction volume of the citrate–phosphate buffer, which was incubated at 30 °C [36]. The reactions were interrupted at different intervals (0, 90, 180 or 240 min) by incubating the mixture at 99 °C for 5 min. The amount of glucose released was determined using the glucose mono-reagent kit (K082-Bioclin, Belo Horizonte, MG, Brazil), based on the reaction of glucose oxidase. For this, the glucose oxidase reagent (200 µl) was added to the reaction sample (50 µl) and incubated at 37 °C for 15 min [36, 37]. The plates were read at 505 nm in the SpectraMax 190™ (Molecular Devices, San Jose, CA, USA). The amount of released reaction product was determined using a standard curve of glucose (10–100 nmol) read under the same conditions as the test samples. From 38 to 46 individual midgut samples were analyzed per strain.

### Proteases assays

To measure protease activity (E.C. 3.4), whole midgut samples were subjected to a continuous assay using three different substrates (Sigma-Aldrich): N-succinyl-Ala-Ala-Phe-7-amido-4-methylcoumarin for chymotrypsin, Z-Phe-Arg-7-amido-4- methylcoumarin for trypsin and-L-Leu-7-amido-4-methylcoumarin for aminopeptidase. Midguts were homogenized in 100 µl saline solution (0.9%). The reaction was composed by an aliquot of midgut sample (20 µl for N-Ala-Ala-Phe and l-Leu; 40 µl for Z-Phe-Arg), a final concentration of 10 µM of each substrate in a 200 µl-final reaction volume of 200 mM Tris–HCl (pH 8), which was incubated at 30 °C for 1 h. The enzymatic assay was based on the continuous detection of the methylcoumarin acid (MCA) released from the substrate hydrolysis, which was recorded at excitation of 380 nm and emission of 460 nm in the microplate reader SpectraMax Gemini XPS™. The amount of product released was calculated from a standard curve (0.01–0.2 nmol) of MCA (Sigma-Aldrich), recorded under the same conditions as the test samples. A total number from 45 to 51 individual midgut samples was analyzed per strain.

### Quantification of lipid reserves

The amount of lipids was quantified in 30 pools of 20 early fourth instar larvae and in 30 newly emerged (20 h) females individually using a vanillin-phosphoric acid colorimetric method which determines total lipids, adapted from previous protocols [38]. Samples (pool of 20 larvae or one female) were prepared by homogenizing the specimens under ice in 200 µl of 2% sodium sulfate. An aliquot of chloroform–methanol (1:1 ratio, 800 µl) was added, the sample was centrifuged (3000 ×*g*, 5 min) at room temperature (RT), and the supernatant was collected. This step was repeated, and the supernatant was collected. Ultrapure water (600 µl) was added to the supernatant collected, and the sample was centrifuged (3000 ×*g*, RT, 5 min). After centrifugation, the sample was separated into two phases, and the bottom phase was collected for lipid analysis. The sample was transferred to a test tube and was heated (90–110 °C) for solvent evaporation. For lipid quantification 200 µl of sulfuric acid (98%) was added to the sample and was incubated at 100 °C for l0 min. Then, vanillin-phosphoric acid (q.s. 5 ml), prepared according to Van Handel [38], was added to the sample and gently mixed. This sample was incubated on ice for 5 min. After that, a 1-ml aliquot of the sample was used to read the absorbance at 525 nm in the UltroSpec2100^™^ (Amersham Biosciences, Amersham, UK). The amount of lipid in the samples was determined using a standard curve (25–300 µg) of commercial soybean oil (Liza^®^ batch L03B, Uberlândia, MG, Brazil), mainly composed of triglycerides, which was read under the same conditions as the test samples.

### Quantification of reducing sugars

The reducing sugars were determined in 15 pools of 40 early fourth instar larvae and 15 pools of five newly emerged (20 h) females, based on Yamada et al. [39]. Samples (pool of 40 larvae or pool of five females) were homogenized under ice with 200 µl of a mixture of methanol, ultrapure water and chloroform (2:1:1 ratio). The samples were incubated (− 30 °C, 30 min) and centrifuged (21,000 ×*g*, 4 °C, 20 min). Then, the pellet was washed with ultrapure ethanol (300 µl) and dried at RT. An aliquot of 400 µl of phosphate saline buffer (0.2X, pH 7.4) with Triton X-100 0.1% was added to the sample and incubated (70 °C, 30 min). After this, the 400-µl sample was divided in a test and a negative control sample, each with 200 µl. The test sample was incubated with amyloglucosidase (0.5 mg/ml final, Sigma-Aldrich) at 60 °C for 30 min. The negative control sample was incubated under the same conditions without the enzyme. After the incubation, dinitrosalicylic acid (500 µl) was added to the samples, and the reaction was stopped by heating (100 °C, 6 min). Each sample (700 µl) was incubated on ice for 15 min. Then, a 400-µl aliquot of the test sample or control sample was read at an absorbance of 540 nm in a 96-well microplate reader Benchmark Plus^™^ (Bio-Rad, Hercules, CA, USA). The absorbance of the samples was converted to the concentration of glucose (Sigma-Aldrich) using a standard curve (0.06–3 mg/ml). The concentration of reducing sugars was determined based on the variation of the glucose concentration observed in the test sample and negative control sample.

### Fertility and fecundity assays

The fertility and fecundity were evaluated using females reared under optimal conditions previously described [28], or under stress conditions. The evaluation under stress was done since it might provide a better resolution to detect changes in biological parameters under such condition, as observed in a previous study [28]. Briefly, as described, under optimal conditions, first instar larvae from one raft (~ 250 eggs) were set in a plastic tray with tap water (1 l), and cat food (0.8 g) was provided. For simulating stress, 600 first instar larvae per tray were maintained under the same conditions described above. Adults from both rearing groups were kept under insectary conditions, and 5-day-old females were fed with defibrinated rabbit blood, as described. Egg rafts from their first gonadotrophic cycle were collected until 24 h after the oviposition. The fertility and fecundity were evaluated at 0, 3, 5 and 8 days post-oviposition (dpo). To assess quiescence during these storage times, egg rafts were kept in a humid chamber (Petri dish, 60 × 15 mm) at RT. For each raft, the egg number was recorded under a stereo microscope. After that, the raft was set in a plastic tray with tap water (1 l) and food (0.1 g). The raft was kept under insectary conditions for larval hatching. Fecundity was determined based on the hatching of first instar larvae within 36 h after the raft was set in the tray. In the assay, each experimental point was carried in duplicate (at least two egg rafts per condition per strain). Therefore, at least 32 eggs rafts were analyzed per assay, and each assay was performed at least twice.

### Statistical analysis

The analysis was performed using GraphPad Prism^™^ 6.0 (San Diego, CA, USA). First, the D’Agostino-Pearson Omnibus K2 normality test was used to determine how far the distribution was from Gaussian in terms of asymmetry and shape. For parametric samples, the outliers were identified by the ROUT method based on the false discovery rate (FDR), Q being established as 1%. For comparison of normally distributed data, the unpaired t-test was used, and significance was considered when *P* < 0.05. For comparison of non-normally distributed data, the Mann-Whitney test was used, and significance was considered when *P* < 0.05. Results are expressed as means and standard deviation (SD).

## Results

### Enzyme activities

**Fig. 1:**
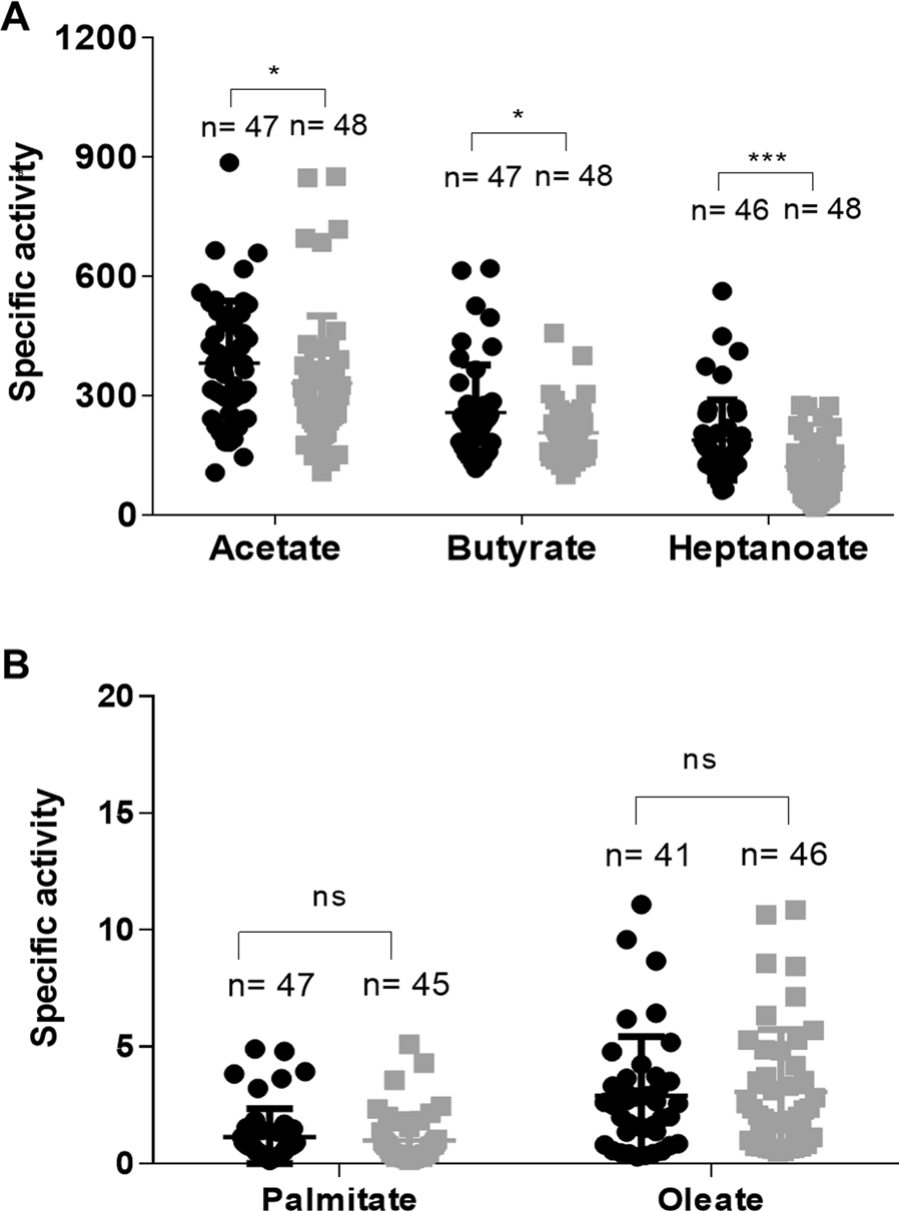
Esterase and lipase specific activity (U/g protein) in individual midguts of *Culex quinquefasciatus* fourth instar larvae from a susceptible (black circles) and Bin-resistant (gray squares) strain, using different substrates. **A** Acetate, butyrate and heptanoate. **B** Palmitate and oleate. The results are the mean and standard deviation of four biological replicates. Mann-Whitney test, statistical differences of ***P < 0.001, *P < 0.01 or *ns* not significant difference

These assays were performed to investigate whether some differential aspects found in the transcriptome of resistant larvae corresponded to altered catalytic activities in those individuals. The activity of esterases and lipases was recorded for larvae from both strains using five substrates differing in chain length (Fig. 1). The specific activity was detected for all substrates, decreasing for acetate, butyrate, heptanoate, oleate and palmitate. The comparison between strains showed that resistant larvae had a significantly lower hydrolytic specific activity (µU/µg protein) compared to susceptible controls for MU-acetate (*R* = 330 ± 20, *S* = 380 ± 20), MU-butyrate (*R* = 206 ± 9, *S* = 250 ± 10) and MU-heptanoate, which showed the most significant reduction (*R* = 120 ± 10, *S* = 180 ± 10) (Fig. 1A). The resistant larvae showed lower specific activity of MU-oleate and higher for MU-palmitate, compared to susceptible larvae, but these differences were not statistically significant (Fig. 1B). When comparing the substrates above with different chain lengths, the hydrolase activity in the larvae midgut was lower for oleate and palmitate, which have longer carbon chain length compared to acetate, butyrate and heptanoate. The results showed that resistant larvae had, in general, reduced hydrolase activities against these substrates compared to the susceptible strain.

**Fig. 2:**
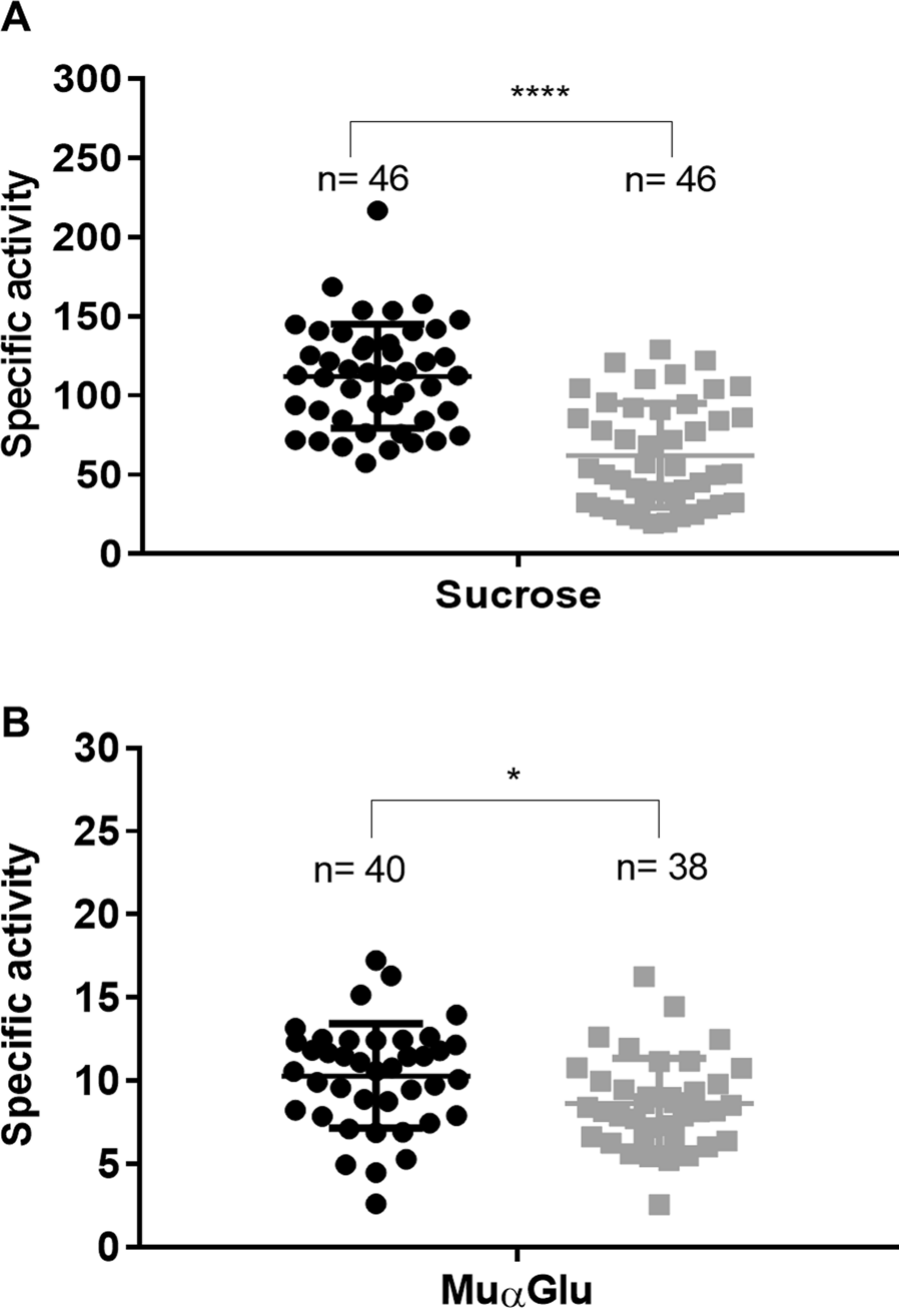
α-Glucosidase-specific activity (U/g) in individual midguts of *Culex quinquefasciatus* fourth instar larvae from a susceptible (black circles) and a Bin-resistant (gray squares). **A** Sucrose. **B** 4-Methylumbelliferyl α-glucopyranoside (MUαGlu). The results are the mean and standard deviation of three biological replicates. Mann-Whitney test, statistical differences of ****P < 0.0001, *P < 0.01

The activity of α-glucosidase was detected in larvae of both strains using a natural (sucrose) and a synthetic (MUαGlu) substrate. The specific activity (U/g protein) was higher on sucrose than on MUαGlu in all samples (Fig. 2). The comparison between strains showed that the activity on sucrose was remarkably lower in resistant individuals (62 ± 5), compared to susceptible ones (112 ± 4) (Fig. 2A). Meanwhile, for the synthetic substrate (MUαGlu) this activity was also lower (8.6 ± 0.4) in resistant larvae compared to susceptible ones (10.3 ± 0.5) (Fig. 2B).

**Fig. 3:**
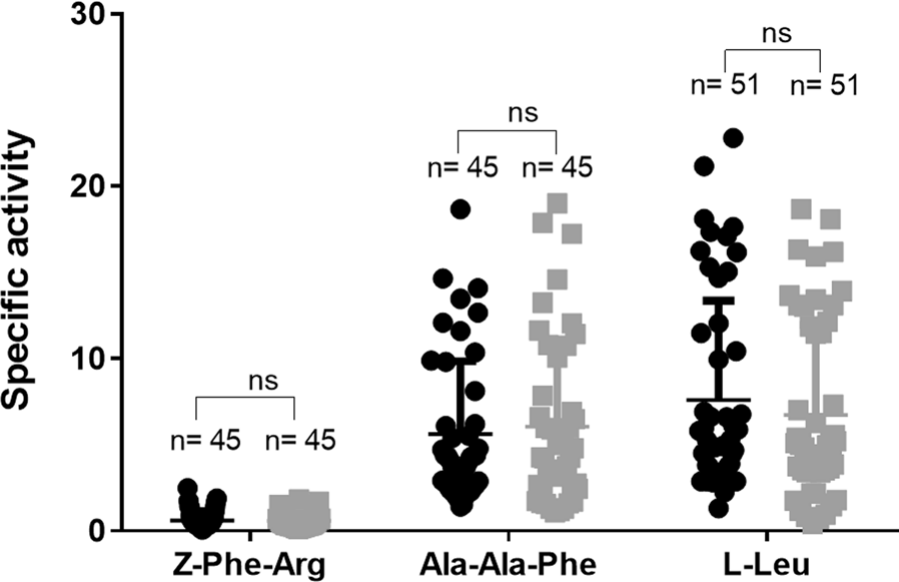
Proteases specific activity (U/g) in individual midguts of *Culex quinquefasciatus* fourth instar larvae from a susceptible (black circles) and a Bin-resistant (gray squares) using substrates for trypsins (Z-Phe-Arg), chymotrypsins (N-Ala-Ala-Phe) and aminopeptidases (l-Leu). The results are the mean and standard deviation of three biological replicates. Mann-Whitney test, no significant differences (ns) were found

The activity of three major proteases found in the larval midgut, trypsin (substrate Z-Phe-Arg-MCA), chymotrypsin (substrate Ala-Ala-Phe-MCA) and aminopeptidase (substrate l-Leu-MCA) was detected in larvae of both strains (Fig. 3). When comparing the hydrolysis rates of these substrates, the specific activities were higher for chymotrypsins and aminopeptidases compared to trypsins. The comparative analyses between strains showed similar activity for all substrates since no statistically significant differences were found as follows: Z-Phe-Arg-MCA (*R* = 0.59 ± 0.06, *S* = 0.60 ± 0.08), Ala-Ala-Phe-MCA (*R* = 6.1 ± 0.7, *S* = 5.6 ± 0.6) and l-Leu-MCA (*R* = 6.7 ± 0.7, *S* = 7.6 ± 0.8) (Fig. 3). The complete dataset of enzyme assays is available in the Additional files 2, 3, 4: Tables S2, S3, S4.

### Energy reserves

**Fig. 4:**
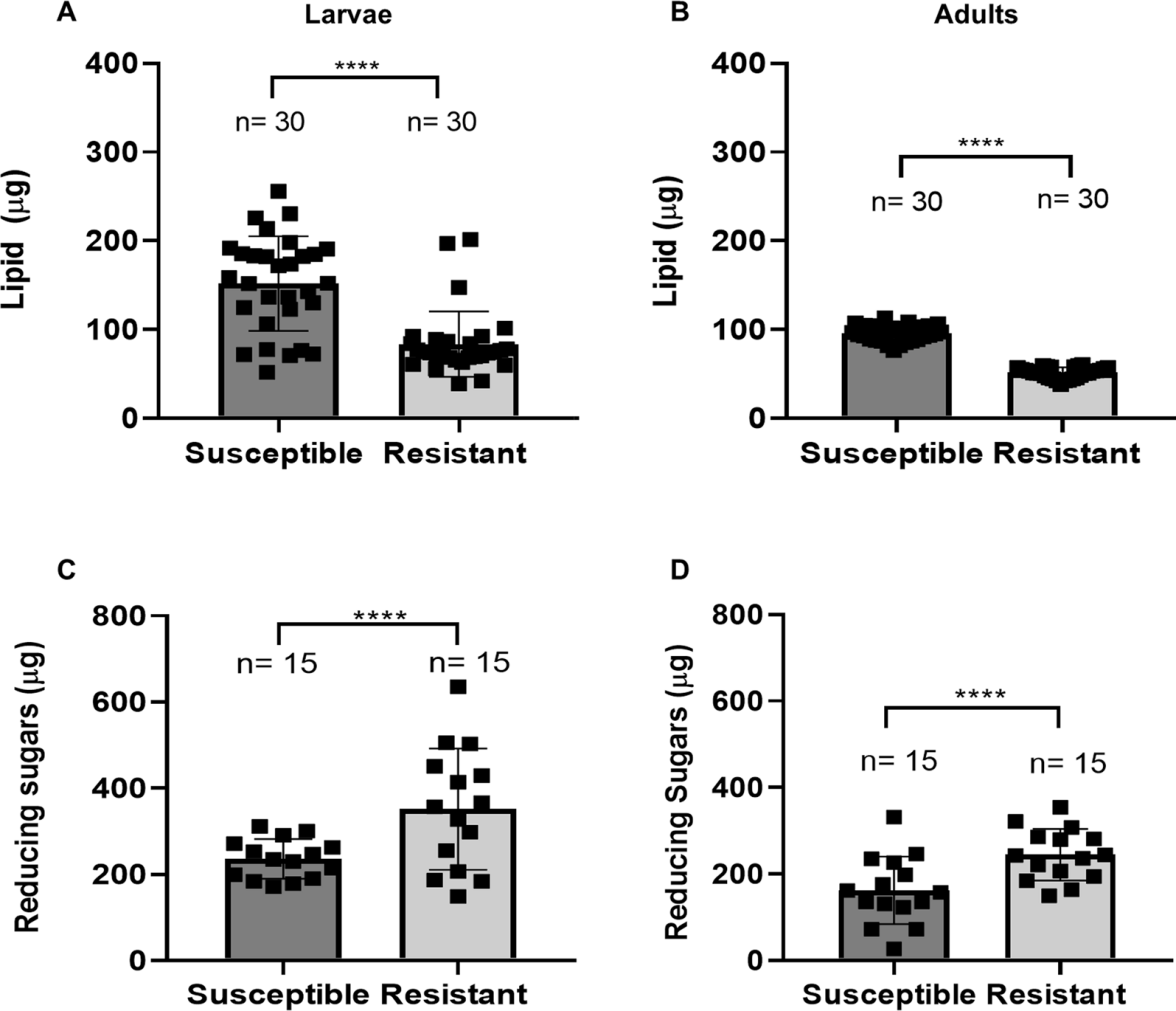
Energy reserves of *Culex quinquefasciatus* from a susceptible and a Bin-resistant strain. **A** Lipid (µg) in pools of 20 fourth instar larvae. **B** Lipid (µg) in adult females. **C** Reducing sugars (µg) in pools of 40 larvae. **D** Reducing sugars (µg) in pools of five females. The results are the mean and standard deviation of biological replicates. Mann-Whitney test, statistical differences of ****P < 0.0001

We quantified the lipids and reducing sugars from larvae and adults to investigate whether the difference in expression of genes related to the metabolism of lipid and carbohydrate that was found between the resistant and susceptible strains would affect the energy resources of these insects (Fig. 4). Pools of resistant larvae (*n* = 20) showed a significantly lower amount (µg) of lipids (72.6 ± 6) compared to pools of susceptible larvae (125.7 ± 9) (Fig. 4A). The lipid reserve (µg) in resistant adults (*n* = 1) was also significantly lower (47.5 ± 1) compared to the susceptible ones (82.3 ± 2) (Fig. 4B). The reserves of reducing sugars (µg) were also different between strains. In this case, pools of resistant larvae (*n* = 40) (351.5 ± 36) and pools of resistant adults (*n* = 5) (245 ± 15) showed a higher amount of reducing sugars compared to the corresponding pools of susceptible larvae (236.3 ± 12) and adults (162 ± 20) (Fig. 4C and D). The dataset showed that resistant individuals exhibit significant alteration in the reserve of both sources as larvae and adults. In general, resistant larvae showed 58% fewer lipids and 33% more reducing sugars reserves than the susceptible ones, while resistant adults showed 58% fewer lipids and 34% more reducing sugars reserves. The dataset of those assays is available in the supplementary information (Additional files 5, 6: Tables S5 and S6).

### Fertility and fecundity

**Fig. 5:**
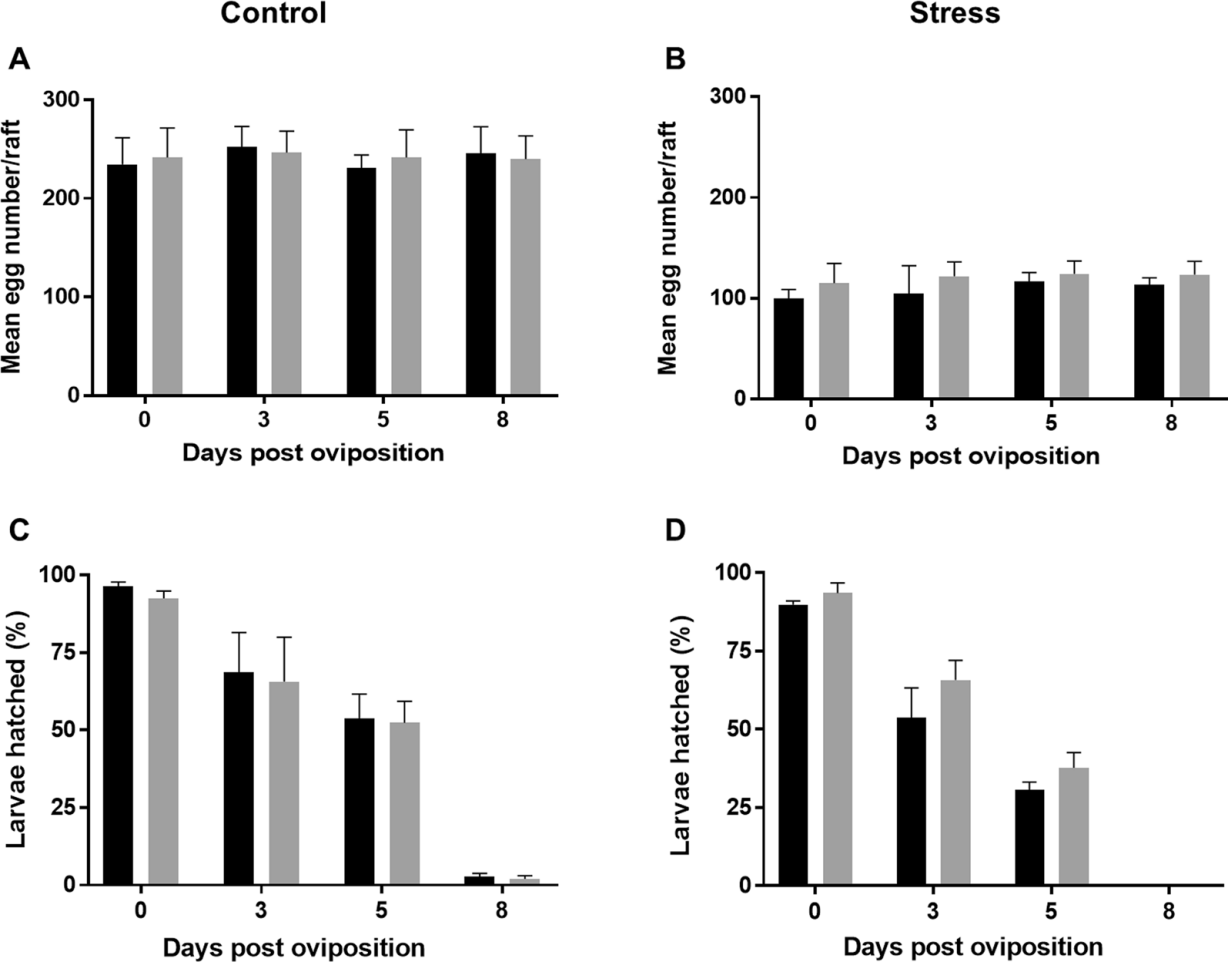
Fertility and fecundity of *Culex quinquefasciatus* females from a susceptible (black) and a Bin-resistant (gray) strain reared under controlled (CF) or stress conditions (EF). Eggs stored in a humid chamber until 8 days post oviposition were analyzed. **A** Mean egg number per raft from CF. **B** Mean egg number per raft from EF. **C** Percentage of larvae hatching per raft from CF. **D** Percentage of larvae hatching per raft from EF. Columns and bars represent the average and standard deviation of at least four experimental points. Unpaired t-test, no significant differences were found

The fertility and fecundity of females were investigated to evaluate whether the changes in lipid and sugar contents found between the strains could impact reproductive aspects related to these reserves. Those parameters were assessed using eggs from resistant and susceptible females reared under optimal or stress conditions. Eggs were analyzed immediately after oviposition and after storage under humid conditions, as described. The mean number of eggs from females reared under optimal conditions on day 0 was similar for susceptible (234 ± 23) and resistant (241 ± 26) individuals, and no statistical differences were observed (Fig. 5A). The mean number of eggs recorded in those rafts stored at 3, 5 and 8 dpo in a humid chamber was also similar for both strains in the different replicates (Fig. 5A). For females reared under stressing conditions, a significant reduction in the mean egg number on day 0 for both susceptible (99 ± 8) and resistant strains (114 ± 17) was recorded (Fig. 5B). These amounts were similar in the batches of both strains analyzed at all time points (Fig. 5B). Meanwhile, the hatching percentage of eggs from females reared under controlled conditions on day 0 was 96 ± 1 and 92 ± 2 for susceptible and resistant strains, respectively, showing high and similar viability for both strains (Fig. 5C). Eggs from those females that were stored during 3 and 5 dpo resulted in first instar larvae but the percentage dropped, similarly for both strains (at 3 dpo *S* = 68 ± 11 and *R* = 65 ± 12; at 5 dpo *S* = 53 ± 6 and *R* = 52 ± 6) (Fig. 5C). For the egg rafts stored for 8 dpo, only 2% of first instar larvae were detected for both strains (Fig. 5C). The hatching percentage of eggs from females reared under stress conditions also showed a decrease, which was statistically similar to the control group (Fig. 5D). The impact of the stress rearing condition on the fertility was observed for females from both strains. The reduction of fecundity was observed in eggs from samples at 3 and 5 dpo for both strains (Fig. 5D), being less marked for the resistant strain, but the differences were not statistically significant. No hatching of eggs stored during 8 dpo was recorded (Fig. 5D). The complete dataset from those assays is presented in Additional file 7: Table S7.

## Discussion

Data from this study showed that a *Cx. quinquefasciatus* strain resistant to the larvicidal Binary toxin from *L. sphaericus* displayed differential metabolic features, as previously indicated by the transcriptomic profile of this strain [29]. The suggestion that the metabolism of lipids may be changed in the resistant strains led us to follow the activity of lipases in these insects, using fluorescent substrates, derived from substituted esters of carboxylic acids with different chain lengths, routinely used for lipase screening and characterization [40, 41]. Our results suggested a predominance of the activity of carboxylesterases, which in general prefer short-chain substrates, over true lipases, which tend to prefer long-chain substrates. However, the substrate specificities of these enzymes have a considerable superposition, especially against water-soluble and very sensitive fluorescent substrates such as MU-esters [42–45]. Notably, increased activity of carboxylesterases can be involved in several examples of *Culex* metabolic resistance to chemical insecticides [46, 47]. However, in RIAB59-resistant larvae, those enzymes displayed a reduced activity, which is not consistent with a role for an increased detoxification of xenobiotics. It is likely that the lower activity of these enzymes on fatty acids, as butyrate and acetate for instance, could be involved with other processes, as further discussed.

The resistant strain showed a significant reduction of lipid storage in both larvae and adults, indicating alterations in the metabolism of lipids. The lower hydrolytic activity in the resistant larvae to catalyze the fatty acids tested could be related to that, but several other repressed transcripts involved in lipid metabolism were also found, including lipases, phospholipases, triacylglycerol lipases, apolipoprotein D, apolipophorin III, fatty acid hydroxylase superfamily, pyruvate desidrogenase and pantetheinase [29]. The repression of transcripts such as lipases and pantetheinases, for instance, could provoke rather a greater lipid accumulation than a reduced one. On the other hand, the 2-hydroxyacyl-CoA lyase 1, which is involved in the digestion of fatty acids, was the top upregulated transcript in RIAB59 larvae [29]. Therefore, it is not possible to determine how the alterations found in the enzyme activities and transcripts could impact the lipid accumulation because of the complexity of the pathways and molecules possibly involved. Nevertheless, our data strongly suggest its relation with the resistance status since the most important factors that could have a direct influence on those reserves, such as diet [48–50] and larval rearing density [51], were kept under control during this study.

Another aspect related to energy reserves was the assessment of α-glucosidase activity and the comparison of the reserve of reducing sugars in resistant RIAB59 individuals, which are characterized by the lack of expression of the Cqm1 α-glucosidases on the midgut [23]. The resistant larvae showed significantly lower α-glucosidase activity using sucrose and MUαGlu as substrates, which contrasted with a previous evaluation of two other Bin-resistant strains that were also characterized by the lack of Cqm1 [28]. Still, they showed similar α-glucosidase activity, using either sucrose or the synthetic substrate [28]. However, the marked increase of reducing sugars, in both resistant larvae and adults, suggested that the metabolism of carbohydrates in these individuals was impacted. The outstanding altered energy reserves in resistant individuals might have important consequences since lipids and sugars are the major sources for mosquito’s development and survival and their metabolisms are intrinsically related [31, 52]. The glycogen is the direct source of glucose for metamorphosis and flight and is also an important source for producing fatty acids by de novo synthesis [31, 52]. This process contributes to the homeostasis of lipids by activating fatty acids synthesis when carbohydrates are available for this purpose. The de novo synthesis of fatty acids can also occur through the incorporation of acetate, which is metabolized into triacylglycerol, as described in insects, including *Aedes aegypti* [53–55].

Another important aspect that could impact the energy reserves is the microbiota of mosquitoes. A recent study showed that *Culex pipiens pipiens* that displayed a reduced microbiota, compared to a control strain, had higher carbohydrate reserves and a lower lipid reserve, indicating the inability of those individuals to convert sugars to accumulate lipids [56]. Valzania et al. [57] also showed that *Ae. aegypti* larvae subjected to bacterial elimination displayed an alteration in their ability to accumulate lipids. The mechanism is not fully understood, but it has been shown that in mammals, the microbiota can play a central role in the production of short-chain fatty acids (e.g. acetate, butyrate and propionate) that act as signaling molecules to modulate the energy metabolism of the host [58]. In our study the reduced ability of the resistant larvae to hydrolyze such short-chain fatty acids could also be implicated in the modulation of lipid accumulation. In parallel, recent studies have raised concerns about the exposure of mosquitoes to microbial larvicides and their impact on their microbiota and, consequently, on the biological processes modulated by the bacterial community [59, 60]. Our resistant *Cx. quinquefasciatus* strain that had been chronically exposed to *L. sphaericus* showed a significant change in the energy reserves. Taking these data together, it is possible to consider that microbiota could also be involved in this process.

Results from this study showed functional data that could be related to some aspects of the differential transcriptomic profile of RIAB59 resistant larvae, but at a limited scale, considering the complexity of energy metabolism. For aminopeptidases, trypsins and chymotrypsins, enzymes that are involved in digestion and insect immunity [61, 62], for instance, no differential activity in resistant larvae was found. This suggests that other forms of regulation of expression might be involved, like post-transcriptional or post-translational mechanisms, that were already described for the regulation of gut protease activities in insect vectors [63]. Alternatively, compensation for repression of some genes might be overcome by overexpression of other gene products in the multigenic families of serine proteases and aminopeptidases [64, 65].

Lipids are essential as energy reserves and play critical roles in immunity and reproduction, as reviewed by Gondim et al. [52]. However, the potential effects of the reduced lipid reserves on egg production and viability of resistant females were not found, although the significant reduced lipid accumulation could severely affect these reproductive parameters, as observed in other studies [66, 67]. The significant reduction of the number of eggs produced by females derived from larvae reared under stress, observed for both strains, seems to be a direct deleterious effect of lower food availability*,* as seen in other studies [68–70]. The fertility was also similar for resistant and susceptible females, but the relatively high rate of larval hatching (~ 50%) of eggs from both strains stored for 5 days in a humid chamber was not expected since *Cx. quinquefasciatus* does not display quiescence [71–73].

The understanding of insecticide resistance in mosquitoes revealed three major mechanisms: changes in the target site, metabolic resistance and changes in the cuticle barrier [74]. In general, changes in the target site result in fitness costs, and metabolic or cuticular resistance can be related to cross-resistance. Our studies on *L. sphaericus* resistance in *Cx. quinquefasciatus* revealed a new and interesting interaction between a target site mutation (*cqm1* with a deletion) and several metabolic changes that resulted in minimal fitness cost, which contrasts with the significant costs often reported for the resistance to chemical insecticides [75, 76]. It would be important to understand if this phenomenon extends to other vectors and active molecules or if this is restricted to the *L. sphaericus*/*Cx. quinquefasciatus* interaction, or to the group of microbial insecticides. From this perspecitve, the microbiota might be an important parameter to be investigated in the future.

## Conclusions

In summary, our dataset showed that a Bin toxin-resistant *Cx. quinquefasciatus* strain displayed significant changes in the activity of esterases and α-glucosidases and a remarkable alteration of energy reserves, in particular lipid accumulation. Although the fecundity and fertility of females were not impacted under the conditions tested, the alterations found might potentially affect other important aspects of mosquito physiology.

### Supplementary Information

**Additional file 1: Table S1.** Differentially expressed genes with log2 foldchange > 1.5 related to the metabolism of lipids which were found in the transcriptome of a *Culex quinquefasciatus* larva from *Lysinibacillus sphaericus* RIAB59 resistant strain compared to a susceptible one. Data from Rezende et al. [29].

**Additional file 2: Table S2.** Dataset of the esterase and lipase activity assays in individual midguts of *Culex quinquefasciatus* early fourth instar larvae from a susceptible and a Bin-resistant strain, using five different substrates. Lipase activity (A; mU/midgut). Protein (P; µg/midgut). Specific activity (SA; U/g protein).

**Additional file 3: Table S3.** Dataset of the α-glucosidase activity assays in individual midguts of *Culex quinquefasciatus* early fourth instar larvae from a susceptible and a Bin-resistant strain using two different substrates. α-Glucosidase activity (A; mU/midgut). Protein (P; µg/midgut). Specific Activity (SA; U/g protein).

**Additional file 4: Table S4.** Dataset of the protease activity assays with substrates for trypsins (Z-Phe-Arg-MCA), chymotrypsins (N-Ala-Ala-Phe-MCA) and aminopeptidases (l-Leu-MCA) in individual midguts of *Culex quinquefasciatus* early fourth instar larvae from a susceptible and a Bin-resistant strain. Protease activity (A; mU/midgut). Protein (P; µg/midgut). Specific activity (SA; U/g protein).

**Additional file 5: Table S5.** Dataset of assays of lipid quantification in pools of early fourth instar larvae (*n* = 20) and individual female samples of *Culex quinquefasciatus *from a susceptible and a Bin-resistant strain.

**Additional file 6: Table S6.** Dataset of assays performed for the quantification of reducing sugars in pools of early fourth instar larvae (*n* = 40) and pools of female (*n* = 5) *Culex quinquefasciatus *from a susceptible and a Bin-resistant strain. Absorbance at 540 nm (Abs). Test sample incubated with amyloglucosidase (T). Control sample incubated without enzyme (C). Final reducing sugars amount (S).

**Additional file 7: Table S7.** Dataset of assays to determine the fecundity and fertility of *Culex quinquefasciatus *females from a susceptible and a Bin-resistant strain under controlled or stressing rearing condition. Days post oviposition (dpo). First instar larvae percentage (LI%). Replicate (R). Standard deviation (SD).

## Data Availability

Raw data from all assays are available in the supplementary tables.

## Abbreviations

Bin: Binary toxin
BSA: Bovine serum albumin
DEGs: Differential expressed genes
DPO: Days post-oviposition
FDR: False discovery rate
GPI: Glycosylphosphatidylinositol
MCA: Methylcoumarin acid
MU: 4-Methylumbelliferyl
MUαGlu: 4-Methylumbelliferyl α-glucopyranoside
R: RIAB59 strain
RT: Room temperature
S: CqSLAB strain
SD: Standard deviation
