# Supplementary material for: A Culex quinquefasciatus strain resistant to the binary toxin from Lysinibacillus sphaericus displays altered enzyme activities and energy reserves
Source: Parasit Vectors. 2023 Aug 9;16:273. doi: 10.1186/s13071-023-05893-z (PMC10413512; doi:10.1186/s13071-023-05893-z)
Supplement: Supplementary file 1 — Additional file 1: Table S1. Differentially expressed genes with log2 foldchange > 1.5 related to the metabolism of lipids which were found in the transcriptome of a Culex quinquefasciatus larva from Lysinibacillus sphaericus RIAB59 resistant strain compared to a susceptible one. Data from Rezende et al. [29]. [file 13071_2023_5893_MOESM1_ESM.pdf]

Additional file 1: Table S1. Differentially expressed genes (log2 foldchange> 1.5) related to the metabolism of lipids which were found in the transcriptome of a *Culex quinquefasciatus* larvae from *Lysinibacillus sphaericus* RIAB59 resistant strain, compared to a susceptible one. Data extracted from [1].

**Gene_id**	**Annotation**	**log2 FoldChange**	***P*-value**
**Downregulated**			
CPIJ017593	pantetheinase precursor	7,13	1,51E-49
CPIJ017592	Vanin-like protein 1 precursor, putative	6,14	1,06E-31
CPIJ009045	Fatty acid hydroxylase superfamily	4,19	1,67E-39
CPIJ015726	apolipoprotein D, putative	4,10	6,54E-13
CPIJ002726	lipase 3 precursor	3,52	8,39E-18
CPIJ014889	pyruvate dehydrogenase	3,47	3,65E-09
CPIJ016451	crotonobetainyl-CoA dehydrogenase	3,39	1,37E-101
CPIJ015727	apolipoprotein D, putative	3,34	4,17E-10
CPIJ004369	glucosyl transferase	3,31	3,74E-12
CPIJ017588	peroxidase precursor	3,13	5,69E-08
CPIJ004230	lipase	2,94	4,80E-71
CPIJ011600	long-chain-fatty-acid coa ligase	2,14	1,13E-20
CPIJ003870	acyl-CoA oxidase	2,04	9,58E-07
CPIJ016639	acetyl-coa synthetase	1,85	2,96E-06
CPIJ016336	esterase B1 precursor	1,75	1,39E-29
CPIJ004141	1-acyl-sn-glycerol-3-phosphate acyltransferase beta	1,68	1,10E-07
CPIJ004227	lipase	1,64	1,12E-47
CPIJ004228	lipase	1,63	5,37E-62
CPIJ018160	lipid storage droplets surface binding protein 2	1,62	3,48E-06
CPIJ000045	carboxylesterase	1,61	2,77E-04
**Upregulated**			
CPIJ014172	2-hydroxyacyl-CoA lyase 1	5,33	1,61E-24
CPIJ000426	acetyl-coenzyme A synthetase	2,34	2,76E-20
CPIJ006434	dihydroxyacetone kinase	1,71	1,12E-18
CPIJ013313	apolipoprotein D, putative	1,60	5,63E-13
CPIJ016763	short-chain dehydrogenase	1,59	3,15E-04

Reference

1. Rezende TMT, Rezende AM, Luz Wallau G, Santos Vasconcelos CR, de-Melo-Neto OP, Silva-Filha M, Romao TP: **A differential transcriptional profile by *Culex quinquefasciatus* larvae resistant to *Lysinibacillus sphaericus* IAB59 highlights genes and pathways associated with the resistance phenotype**. *Parasit Vectors* 2019, **12**(1):407.
