# Supplementary material for: A Culex quinquefasciatus strain resistant to the binary toxin from Lysinibacillus sphaericus displays altered enzyme activities and energy reserves
Source: Parasit Vectors. 2023 Aug 9;16:273. doi: 10.1186/s13071-023-05893-z (PMC10413512; doi:10.1186/s13071-023-05893-z)
Supplement: Supplementary file 2 — Additional file 2: Table S2. Dataset of the esterase and lipase activity assays in individual midguts of Culex quinquefasciatus early fourth instar larvae from a susceptible and a Bin-resistant strain, using five different substrates. Lipase activity (A; mU/midgut). Protein (P; µg/midgut). Specific activity (SA; U/g protein). [file 13071_2023_5893_MOESM2_ESM.pdf]

**Additional file 2: Table S2.** Dataset of the esterase and lipase activity assays in individual midguts of *Culex quinquefasciatus* early fourth instar larvae from a susceptible and a Bin-resistant strain, using five substrates. Lipase activity (A; mU/midgut). Protein (P; µg/midgut). Specific Activity (SA; U/g protein).

| **Susceptible** | | | | | | | | | | | | | | | |
|---|---|---|---|---|---|---|---|---|---|---|---|---|---|---|---|
| | **Acetate** | | | **Butyrate** | | | **Heptanoate** | | | **Palmitate** | | | **Oleate** | | |
| **N** | **A** | **P** | **SA** | **A** | **P** | **SA** | **A** | **P** | **SA** | **A** | **P** | **SA** | **A** | **P** | **SA** |
| 1 | 7.446 | 11.192 | 665.341 | 4.775 | 20.489 | 233.073 | 2.475 | 19.842 | 124.717 | 0.062 | 33.316 | 1.856 | 0.047 | 7.608 | 6.194 |
| 2 | 7.128 | 15.711 | 453.707 | 4.068 | 17.048 | 238.635 | 2.951 | 19.325 | 152.692 | 0.122 | 37.712 | 3.226 | 0.034 | 3.892 | 8.677 |
| 3 | 7.767 | 34.920 | 222.436 | 4.491 | 18.318 | 245.188 | 2.999 | 19.653 | 152.580 | 0.036 | 31.386 | 1.137 | 0.023 | 4.477 | 5.185 |
| 4 | 8.857 | 9.996 | 886.076 | 4.632 | 17.055 | 271.606 | 2.723 | 14.487 | 187.957 | 0.049 | 32.791 | 1.500 | 0.022 | 2.019 | 11.099 |
| 5 | 8.049 | 15.780 | 510.099 | 5.126 | 21.921 | 233.836 | 3.180 | 21.725 | 146.366 | 0.091 | 18.487 | 4.907 | 0.025 | 6.988 | 3.525 |
| 6 | 7.914 | 21.643 | 365.650 | 4.676 | 18.502 | 252.739 | 3.288 | 7.984 | 411.777 | 0.077 | 20.006 | 3.845 | 0.020 | 7.465 | 2.622 |
| 7 | 6.739 | 12.721 | 529.762 | 4.606 | 18.460 | 249.504 | 4.457 | 26.267 | 169.697 | 0.088 | 22.367 | 3.946 | 0.014 | 0.862 | 16.665 |
| 8 | 6.364 | 18.029 | 353.004 | 4.671 | 30.407 | 153.604 | 3.404 | 23.503 | 144.844 | 0.084 | 54.136 | 1.549 | 0.019 | 7.433 | 2.512 |
| 9 | 6.629 | 10.724 | 618.180 | 3.945 | 24.864 | 158.647 | 3.644 | 22.038 | 165.370 | 0.089 | 52.623 | 1.696 | 0.028 | 17.724 | 1.584 |
| 10 | 7.405 | 16.716 | 443.003 | 3.837 | 15.712 | 244.195 | 3.906 | 38.035 | 102.687 | 0.088 | 24.117 | 3.645 | 0.073 | 11.395 | 6.433 |
| 11 | 6.676 | 14.025 | 476.022 | 3.982 | 20.028 | 198.803 | 3.169 | 51.471 | 61.573 | 0.097 | 20.197 | 4.807 | 0.069 | 26.155 | 2.655 |
| 12 | 7.031 | 16.629 | 422.795 | 4.786 | 9.631 | 496.896 | 3.881 | 17.698 | 219.272 | 3.078 | 20.981 | 0.927 | 0.050 | 11.775 | 4.246 |
| 13 | 6.568 | 12.218 | 537.567 | 3.078 | 17.513 | 223.210 | 3.078 | 16.229 | 189.632 | 3.276 | 12.739 | 1.356 | 0.067 | 18.303 | 3.659 |
| 14 | 5.394 | 22.523 | 239.503 | 3.276 | 25.295 | 115.855 | 3.276 | 26.730 | 122.561 | 2.949 | 21.588 | 0.722 | 0.017 | 20.915 | 0.808 |
| 15 | 5.544 | 22.902 | 242.071 | 2.949 | 27.079 | 127.067 | 2.949 | 17.259 | 170.852 | 2.907 | 26.857 | 0.593 | 0.066 | 17.626 | 3.739 |
| 16 | 5.593 | 17.763 | 314.861 | 2.907 | 26.429 | 130.271 | 2.907 | 20.225 | 143.717 | 3.012 | 23.140 | 0.811 | 0.044 | 21.927 | 2.012 |
| 17 | 6.712 | 22.957 | 292.366 | 3.012 | 20.511 | 183.190 | 3.012 | 20.097 | 149.892 | 3.281 | 25.432 | 0.691 | 0.027 | 19.693 | 1.364 |
| 18 | 6.318 | 59.457 | 106.263 | 3.281 | 16.722 | 206.788 | 3.281 | 29.750 | 110.284 | 2.893 | 27.406 | 0.548 | 0.031 | 20.430 | 1.528 |
| 19 | 6.216 | 24.446 | 254.278 | 2.893 | 22.564 | 169.359 | 2.893 | 18.149 | 159.399 | 3.160 | 19.602 | 0.862 | 0.009 | 17.140 | 0.549 |
| 20 | 5.048 | 24.455 | 206.438 | 3.160 | 25.644 | 148.620 | 3.160 | 19.184 | 164.711 | 2.822 | 21.412 | 0.810 | 0.007 | 15.843 | 0.437 |
| 21 | 6.134 | 42.246 | 145.197 | 2.822 | 21.299 | 148.896 | 2.822 | 43.192 | 65.347 | 3.304 | 12.838 | 1.432 | 0.011 | 26.275 | 0.433 |
| 22 | 6.183 | 25.515 | 242.313 | 3.304 | 19.610 | 201.954 | 3.304 | 22.825 | 144.736 | 3.159 | 37.810 | 0.451 | 0.009 | 11.178 | 0.769 |

| | | | | | | | | | | | | | | | |
|---|---|---|---|---|---|---|---|---|---|---|---|---|---|---|---|
| 23 | 5.891 | 30.955 | 190.304 | 3.159 | 21.256 | 193.440 | 3.159 | 22.553 | 140.085 | 2.896 | 23.247 | 0.756 | 0.031 | 15.716 | 1.966 |
| 24 | 6.130 | 16.111 | 380.452 | 2.896 | 17.087 | 218.317 | 2.896 | 36.637 | 79.043 | 0.011 | 21.632 | 0.495 | 0.020 | 14.935 | 1.371 |
| 25 | 5.750 | 14.499 | 396.619 | 3.848 | 13.789 | 279.078 | 3.311 | 16.928 | 195.562 | 0.007 | 14.742 | 0.473 | 0.033 | 6.948 | 4.787 |
| 26 | 7.212 | 22.105 | 326.249 | 3.847 | 7.319 | 525.646 | 2.834 | 13.767 | 205.872 | 0.009 | 12.481 | 0.736 | 0.009 | 18.978 | 0.455 |
| 27 | 7.453 | 11.313 | 658.796 | 4.518 | 11.436 | 395.073 | 2.900 | 15.860 | 182.819 | 0.007 | 14.811 | 0.482 | 0.008 | 26.194 | 0.297 |
| 28 | 7.269 | 15.911 | 456.861 | 4.789 | 16.789 | 285.270 | 2.730 | 10.713 | 254.870 | 0.007 | 19.791 | 0.346 | 0.009 | 17.133 | 0.553 |
| 29 | 6.981 | 13.803 | 505.746 | 4.740 | 7.712 | 614.631 | 2.706 | 10.579 | 255.795 | 0.007 | 9.093 | 0.754 | 0.010 | 27.370 | 0.359 |
| 30 | 5.662 | 10.626 | 532.871 | 4.557 | 16.476 | 276.595 | 2.507 | 7.117 | 352.255 | 0.007 | 19.795 | 0.344 | 0.062 | 18.504 | 3.328 |
| 31 | 5.586 | 11.566 | 482.988 | 4.530 | 7.309 | 619.869 | 2.567 | 4.562 | 562.754 | 0.006 | 8.454 | 0.673 | 0.034 | 17.101 | 1.990 |
| 32 | 6.912 | 12.368 | 558.890 | 4.601 | 10.871 | 423.208 | 2.629 | 5.854 | 449.016 | 0.009 | 11.918 | 0.751 | 0.031 | 35.679 | 0.858 |
| 33 | 6.994 | 12.926 | 541.054 | 4.434 | 12.137 | 365.313 | 2.705 | 7.246 | 373.311 | 0.011 | 11.846 | 0.906 | 0.062 | 35.987 | 1.723 |
| 34 | 6.007 | 14.092 | 426.264 | 4.604 | 10.575 | 435.385 | 3.200 | 12.035 | 265.879 | 0.011 | 11.428 | 0.946 | 0.095 | 30.192 | 3.162 |
| 35 | 6.558 | 16.622 | 394.552 | 4.701 | 14.144 | 332.331 | 3.160 | 17.946 | 176.096 | 0.010 | 11.874 | 0.840 | 0.042 | 25.593 | 1.640 |
| 36 | 7.480 | 20.536 | 364.265 | 4.697 | 20.584 | 228.203 | 3.483 | 17.760 | 196.102 | 0.007 | 15.848 | 0.466 | 0.085 | 8.835 | 9.585 |
| 37 | 7.481 | 41.105 | 181.987 | 4.680 | 22.598 | 207.085 | 2.644 | 20.810 | 127.057 | 0.006 | 28.315 | 0.195 | 0.063 | 22.170 | 2.853 |
| 38 | 7.351 | 24.310 | 302.399 | 4.984 | 25.369 | 196.464 | 2.868 | 27.804 | 103.138 | 0.007 | 9.846 | 0.759 | 0.054 | 17.062 | 3.175 |
| 39 | 7.209 | 18.798 | 383.482 | 4.714 | 35.496 | 132.793 | 2.725 | 22.468 | 121.266 | 0.006 | 21.431 | 0.296 | 0.083 | 32.183 | 2.571 |
| 40 | 7.340 | 33.387 | 219.847 | 4.785 | 20.610 | 232.188 | 2.655 | 20.941 | 126.772 | 0.005 | 24.583 | 0.206 | 0.104 | 35.525 | 2.917 |
| 41 | 7.109 | 24.095 | 295.021 | 4.704 | 20.113 | 233.872 | 2.302 | 11.222 | 205.098 | 0.005 | 32.080 | 0.147 | 0.119 | 43.854 | 2.711 |
| 42 | 7.387 | 18.100 | 408.122 | 4.687 | 24.060 | 194.785 | 2.914 | 24.806 | 117.487 | 0.008 | 25.941 | 0.317 | | | |
| 43 | 7.289 | 23.580 | 309.134 | 4.640 | 25.213 | 184.038 | 2.593 | 9.686 | 267.647 | 0.006 | 21.546 | 0.271 | | | |
| 44 | 7.449 | 23.594 | 315.710 | 4.742 | 21.569 | 219.845 | 2.688 | 13.450 | 199.831 | 0.008 | 26.513 | 0.287 | | | |
| 45 | 7.194 | 24.031 | 299.369 | 4.927 | 23.751 | 207.428 | 2.851 | 20.769 | 137.247 | 0.007 | 25.414 | 0.294 | | | |
| 46 | 7.415 | 24.471 | 303.011 | 4.827 | 19.939 | 242.077 | 2.750 | 24.303 | 113.147 | 0.007 | 31.282 | 0.227 | | | |
| 47 | 7.186 | 39.208 | 183.277 | 4.727 | 19.849 | 238.153 | | | | 0.007 | 20.579 | 0.330 | | | |

**Resistant**

| | **Acetate** | | | **Butyrate** | | | **Heptanoate** | | | **Palmitate** | | | **Oleate** | | |
|---|---|---|---|---|---|---|---|---|---|---|---|---|---|---|---|
| **N** | **A** | **P** | **SA** | **A** | **P** | **SA** | **A** | **P** | **SA** | **A** | **P** | **SA** | **A** | **P** | **SA** |

| | | | | | | | | | | | | | | | |
|---|---|---|---|---|---|---|---|---|---|---|---|---|---|---|---|
| 1 | 7.278 | 8.588 | 847.538 | 4.906 | 21.755 | 225.500 | 0.628 | 12.074 | 52.019 | 0.064 | 41.804 | 1.540 | 0.050 | 9.359 | 5.295 |
| 2 | 7.619 | 30.939 | 246.266 | 4.727 | 27.740 | 170.407 | 0.612 | 13.311 | 45.980 | 0.054 | 29.904 | 1.801 | 0.056 | 11.557 | 4.849 |
| 3 | 7.151 | 16.669 | 429.033 | 4.594 | 25.625 | 179.277 | 0.638 | 17.284 | 36.923 | 0.072 | 30.753 | 2.336 | 0.062 | 33.250 | 1.855 |
| 4 | 7.691 | 22.393 | 343.465 | 4.590 | 15.094 | 304.072 | 0.613 | 12.588 | 48.725 | 0.054 | 12.594 | 4.302 | 0.050 | 27.667 | 1.806 |
| 5 | 7.563 | 23.790 | 317.917 | 4.907 | 22.989 | 213.469 | 0.585 | 14.580 | 40.107 | 0.051 | 27.894 | 1.834 | 0.035 | 10.556 | 3.327 |
| 6 | 7.736 | 11.119 | 695.690 | 4.731 | 32.112 | 147.342 | 0.573 | 13.061 | 43.884 | 0.053 | 21.521 | 2.451 | 0.050 | 25.615 | 1.935 |
| 7 | 7.115 | 21.897 | 324.913 | 4.931 | 17.108 | 288.223 | 0.591 | 32.122 | 18.402 | 0.057 | 27.952 | 2.028 | 0.046 | 22.566 | 2.031 |
| 8 | 7.372 | 35.689 | 206.568 | 4.961 | 23.284 | 213.075 | 0.601 | 11.245 | 53.459 | 0.094 | 18.352 | 5.101 | 0.062 | 14.828 | 4.195 |
| 9 | 5.921 | 19.981 | 296.321 | 4.751 | 21.343 | 222.582 | 0.561 | 21.315 | 26.300 | 0.061 | 17.130 | 3.572 | 0.041 | 25.341 | 1.637 |
| 10 | 7.090 | 8.332 | 850.932 | 5.104 | 26.789 | 190.534 | 0.580 | 14.230 | 40.737 | 0.022 | 24.962 | 0.895 | 0.046 | 37.791 | 1.209 |
| 11 | 7.404 | 30.912 | 239.515 | 4.783 | 21.738 | 220.054 | 0.561 | 17.302 | 32.398 | 0.020 | 18.836 | 1.039 | 0.065 | 31.311 | 2.074 |
| 12 | 7.035 | 26.477 | 265.720 | 4.889 | 20.510 | 238.380 | 0.577 | 25.757 | 22.394 | 0.018 | 23.892 | 0.744 | 0.053 | 10.046 | 5.286 |
| 13 | 5.678 | 13.469 | 421.583 | 4.452 | 31.079 | 143.237 | 3.174 | 23.408 | 135.597 | 0.020 | 26.394 | 0.769 | 0.017 | 11.434 | 1.521 |
| 14 | 5.777 | 38.866 | 148.631 | 2.991 | 23.975 | 124.757 | 3.038 | 13.748 | 220.960 | 0.019 | 21.084 | 0.923 | 0.023 | 2.764 | 8.435 |
| 15 | 6.178 | 24.481 | 252.363 | 4.013 | 20.972 | 191.370 | 2.918 | 19.514 | 149.523 | 0.020 | 30.356 | 0.645 | 0.036 | 3.348 | 10.659 |
| 16 | 6.213 | 24.406 | 254.561 | 4.005 | 25.060 | 159.831 | 3.301 | 22.292 | 148.068 | 0.020 | 25.593 | 0.765 | 0.052 | 4.773 | 10.860 |
| 17 | 9.938 | 27.937 | 355.733 | 3.844 | 21.080 | 182.335 | 3.295 | 16.709 | 197.183 | 0.020 | 15.069 | 1.351 | 0.017 | 14.066 | 1.227 |
| 18 | 5.847 | 54.378 | 107.522 | 3.745 | 15.992 | 234.206 | 3.406 | 22.593 | 150.772 | 0.018 | 22.195 | 0.801 | 0.019 | 3.409 | 5.706 |
| 19 | 6.307 | 47.136 | 133.796 | 3.485 | 20.569 | 169.430 | 2.961 | 24.008 | 123.346 | 0.019 | 22.774 | 0.816 | 0.015 | 6.306 | 2.360 |
| 20 | 5.900 | 16.087 | 366.785 | 3.506 | 20.266 | 172.996 | 2.898 | 10.528 | 275.239 | 0.019 | 19.511 | 0.973 | 0.031 | 4.377 | 7.135 |
| 21 | 5.746 | 28.514 | 201.527 | 3.306 | 23.967 | 137.937 | 2.123 | 25.470 | 83.350 | 0.019 | 8.869 | 2.160 | 0.008 | 1.285 | 6.348 |
| 22 | 5.610 | 16.359 | 342.930 | 4.075 | 17.727 | 229.882 | 3.228 | 17.622 | 183.175 | 0.010 | 21.407 | 0.479 | 0.025 | 2.887 | 8.576 |
| 23 | 5.877 | 38.845 | 151.296 | 3.948 | 15.371 | 256.838 | 5.256 | 19.197 | 273.798 | 0.008 | 34.467 | 0.241 | 0.007 | 22.133 | 0.726 |
| 24 | 5.782 | 15.856 | 364.672 | 3.799 | 12.478 | 304.421 | 3.139 | 12.791 | 245.414 | 0.010 | 23.879 | 0.408 | 0.016 | 30.152 | 0.649 |
| 25 | 7.210 | 18.453 | 390.744 | 4.835 | 23.159 | 208.788 | 3.271 | 29.570 | 110.625 | 0.009 | 27.480 | 0.318 | 0.020 | 35.476 | 0.744 |
| 26 | 7.318 | 15.813 | 462.799 | 4.503 | 16.991 | 264.997 | 2.794 | 16.680 | 167.482 | 0.011 | 30.793 | 0.342 | 0.026 | 29.158 | 0.722 |
| 27 | 8.012 | 23.571 | 339.893 | 4.979 | 10.907 | 456.488 | 3.196 | 24.422 | 130.868 | 0.010 | 35.362 | 0.273 | 0.021 | 28.053 | 0.992 |
| 28 | 7.570 | 38.000 | 199.216 | 5.118 | 23.928 | 213.893 | 3.251 | 31.791 | 102.261 | 0.009 | 24.816 | 0.367 | 0.028 | 22.243 | 0.709 |

| | | | | | | | | | | | | | | | |
|---|---|---|---|---|---|---|---|---|---|---|---|---|---|---|---|
| 29 | 7.185 | 28.490 | 252.204 | 4.772 | 17.862 | 267.153 | 2.654 | 17.233 | 154.020 | 0.009 | 21.736 | 0.407 | 0.016 | 45.673 | 0.488 |
| 30 | 6.872 | 18.299 | 375.517 | 4.840 | 27.455 | 176.273 | 2.688 | 17.220 | 156.074 | 0.010 | 21.401 | 0.457 | 0.022 | 23.080 | 0.621 |
| 31 | 7.160 | 17.149 | 417.526 | 4.937 | 21.864 | 225.794 | 2.587 | 9.495 | 272.508 | 0.010 | 25.396 | 0.385 | 0.014 | 15.399 | 0.520 |
| 32 | 7.247 | 21.456 | 337.747 | 4.771 | 11.919 | 400.319 | 2.596 | 11.530 | 225.179 | 0.010 | 30.331 | 0.330 | 0.008 | 30.150 | 1.124 |
| 33 | 7.301 | 28.827 | 253.267 | 4.861 | 20.414 | 238.132 | 2.920 | 15.979 | 182.760 | 0.009 | 21.521 | 0.409 | 0.034 | 35.448 | 0.824 |
| 34 | 7.494 | 10.939 | 685.082 | 4.974 | 20.218 | 246.032 | 3.350 | 25.518 | 131.274 | 0.011 | 25.442 | 0.418 | 0.029 | 17.977 | 0.711 |
| 35 | 7.433 | 27.476 | 270.514 | 5.333 | 53.194 | 100.252 | 3.032 | 23.249 | 130.412 | 0.006 | 18.580 | 0.348 | 0.013 | 17.943 | 1.729 |
| 36 | 7.015 | 9.762 | 718.649 | 5.136 | 43.462 | 118.166 | 2.744 | 22.461 | 122.188 | 0.007 | 22.665 | 0.308 | 0.031 | 20.798 | 3.554 |
| 37 | 6.771 | 29.343 | 230.774 | 4.945 | 31.473 | 157.127 | 2.520 | 26.579 | 94.815 | 0.007 | 22.369 | 0.319 | 0.074 | 28.898 | 1.712 |
| 38 | 6.952 | 23.243 | 299.115 | 4.752 | 35.780 | 132.801 | 3.092 | 31.861 | 97.047 | 0.007 | 18.765 | 0.385 | 0.049 | 15.655 | 2.308 |
| 39 | 7.273 | 24.951 | 291.470 | 4.840 | 33.239 | 145.629 | 2.871 | 26.709 | 107.476 | 0.008 | 31.320 | 0.244 | 0.036 | 26.669 | 1.728 |
| 40 | 6.792 | 35.153 | 193.202 | 4.778 | 25.925 | 184.285 | 2.902 | 32.742 | 88.637 | 0.006 | 33.922 | 0.163 | 0.046 | 20.934 | 2.581 |
| 41 | 6.848 | 33.445 | 204.748 | 4.934 | 25.699 | 191.976 | 2.825 | 36.448 | 77.519 | 0.007 | 30.829 | 0.234 | 0.054 | 24.552 | 2.827 |
| 42 | 7.050 | 40.153 | 175.586 | 4.805 | 28.007 | 171.562 | 2.894 | 34.030 | 85.040 | 0.008 | 33.350 | 0.232 | 0.069 | 22.695 | 4.867 |
| 43 | 7.009 | 26.717 | 262.357 | 5.375 | 26.888 | 199.922 | 2.587 | 21.465 | 120.543 | 0.007 | 35.490 | 0.194 | 0.110 | 24.728 | 3.543 |
| 44 | 7.487 | 27.229 | 274.967 | 5.060 | 32.937 | 153.642 | 2.795 | 27.779 | 100.625 | 0.008 | 23.921 | 0.333 | 0.088 | 24.084 | 3.730 |
| 45 | 7.060 | 23.266 | 303.454 | 4.840 | 27.840 | 173.844 | 2.895 | 31.128 | 92.998 | 0.008 | 28.143 | 0.283 | 0.090 | 24.220 | 2.368 |
| 46 | 7.196 | 23.845 | 301.764 | 4.936 | 29.736 | 165.989 | 2.528 | 22.760 | 111.053 | | | | 0.057 | 18.820 | 3.128 |
| 47 | 6.911 | 29.267 | 236.150 | 5.210 | 27.721 | 187.940 | 2.758 | 30.170 | 91.425 | | | | | | |
| 48 | 6.960 | 25.639 | 271.456 | 4.865 | 23.908 | 203.498 | 2.386 | 11.337 | 210.426 | | | | | | |
