# Supplementary material for: A Culex quinquefasciatus strain resistant to the binary toxin from Lysinibacillus sphaericus displays altered enzyme activities and energy reserves
Source: Parasit Vectors. 2023 Aug 9;16:273. doi: 10.1186/s13071-023-05893-z (PMC10413512; doi:10.1186/s13071-023-05893-z)
Supplement: Supplementary file 3 — Additional file 3: Table S3. Dataset of the α-glucosidase activity assays in individual midguts of Culex quinquefasciatus early fourth instar larvae from a susceptible and a Bin-resistant strain using two different substrates. α-Glucosidase activity (A; mU/midgut). Protein (P; µg/midgut). Specific Activity (SA; U/g protein). [file 13071_2023_5893_MOESM3_ESM.pdf]

Additional file 3: Table S3. Dataset of the α-glucosidase activity assays in individual midguts of *Culex quinquefasciatus* early fourth instar larvae from a susceptible and a Bin-resistant strain using different substrates. Glucosidase activity (A; mU/midgut). Protein (P; µg/midgut). Specific Activity (SA; U/g protein).

**Susceptible**						
**N**	**MUαGlu**			**Sucrose**		
	**A**	**P**	**SA**	**A**	**P**	**SA**
1	0.493	45.704	10.778	4.152	54.682	75.939
2	0.293	30.994	9.466	1.013	14.986	67.583
3	0.480	37.931	12.642	3.162	44.241	71.471
4	0.563	45.304	12.429	4.314	38.160	113.056
5	0.543	35.823	15.158	4.907	52.257	93.899
6	0.569	45.497	12.508	3.217	49.099	65.515
7	0.151	21.277	7.103	2.270	21.492	105.611
8	0.634	36.790	17.226	3.283	57.180	57.419
9	0.596	51.872	11.483	3.167	27.529	115.030
10	0.256	26.274	9.732	2.805	27.536	101.864
11	0.469	40.823	11.491	3.208	20.312	157.915
12	0.176	22.249	7.915	2.365	18.407	128.482
13	0.119	26.425	4.485	3.533	30.843	147.924
14	0.187	37.656	4.960	2.083	29.255	139.671
15	0.089	34.113	2.611	2.794	29.418	124.256
16	0.196	18.484	10.583	4.162	45.956	112.843
17	0.205	29.663	6.916	4.652	54.750	127.433
18	0.173	20.950	8.235	2.723	23.339	111.427
19	0.118	15.024	7.866	3.711	28.219	112.607
20	0.219	22.149	9.905	2.054	16.379	168.717
21	0.190	25.547	7.451	2.932	40.782	76.453
22	0.151	21.913	6.889	3.197	24.047	144.832
23	0.262	29.482	8.886	3.910	18.035	121.435
24	0.283	23.284	12.171	2.280	16.195	70.008
25	0.210	17.735	11.849	2.344	15.232	74.640
26	0.400	32.215	12.428	3.110	21.897	131.503
27	0.112	21.197	5.277	4.737	33.708	125.427
28	0.381	37.768	10.091	2.572	21.151	71.883
29	0.472	44.649	10.561	3.451	33.078	132.937
30	0.464	39.748	11.680	3.237	21.083	216.813
31	0.461	36.985	12.475	2.119	23.443	140.771
32	0.327	28.585	11.451	2.460	26.208	153.859
33	0.244	25.384	9.594	2.400	28.456	142.026
34	0.629	53.196	11.820	4.147	28.035	140.535
35	0.533	40.469	13.165	3.323	23.789	121.594
36	0.276	31.567	8.758	3.201	25.761	104.320
37	0.468	42.118	11.104	3.628	32.148	153.553
38	0.496	35.580	13.952	3.228	25.329	90.402
39	0.451	36.543	12.329	3.974	35.666	93.871

40	0.492	30.184	16.311	4.964	44.079	84.324
41				5.382	31.897	114.544
42				1.872	24.488	71.205
43				5.295	36.560	94.988
44				3.390	27.920	90.559
45				4.111	58.721	84.963
46				3.115	41.727	116.689

	**Resistant**					
**N**	**MUαGlu**			**Sucrose**		
	**A**	**P**	**SA**	**A**	**P**	**SA**
1	0.417	66.476	6.268	2.517	19.506	129.055
2	0.290	43.824	6.626	1.082	12.560	86.144
3	0.402	46.985	8.546	2.975	32.775	90.783
4	0.156	27.861	5.600	1.947	16.092	120.979
5	0.600	55.674	10.783	3.206	41.201	77.804
6	0.352	55.148	6.388	3.397	35.486	95.718
7	0.265	43.937	6.036	2.455	33.962	72.288
8	0.227	41.796	5.437	1.852	41.093	45.067
9	0.357	43.834	8.143	2.711	24.569	110.353
10	0.253	37.684	6.704	2.755	26.430	104.224
11	0.161	20.901	7.712	2.267	26.943	84.153
12	0.343	36.680	9.348	1.112	20.063	55.446
13	0.293	37.086	7.898	4.357	50.901	27.904
14	0.241	30.586	7.895	2.666	25.474	40.615
15	0.295	27.402	10.762	3.729	40.359	50.165
16	0.242	33.040	7.321	4.803	45.319	41.709
17	0.311	34.548	9.011	4.603	48.624	54.082
18	0.377	39.841	9.464	4.156	36.690	57.015
19	0.320	28.570	11.199	1.089	36.741	32.311
20	0.260	30.902	8.407	0.697	35.118	50.615
21	0.351	29.335	11.965	3.911	32.027	39.178
22	0.240	35.816	6.694	1.325	38.307	77.458
23	0.317	32.256	9.823	0.881	40.226	72.216
24	0.452	27.775	16.256	0.777	33.712	28.461
25	0.397	27.471	14.459	0.880	27.242	68.721
26	0.281	25.134	11.170	1.252	35.713	29.650
27	0.236	92.110	2.565	1.507	60.957	19.858
28	0.244	46.775	5.224	1.006	41.292	122.131
29	0.447	54.820	8.155	2.024	40.613	34.588
30	0.289	33.365	8.647	1.107	58.170	21.900
31	0.256	31.290	8.197	1.056	33.923	23.047
32	0.484	38.713	12.492	1.273	31.858	32.300
33	0.245	34.052	7.181	1.853	39.634	35.061
34	0.353	27.994	12.626	1.598	57.264	24.730
35	0.265	48.477	5.475	1.659	40.837	24.351
36	0.275	31.639	8.698	3.341	66.600	49.841

37	0.267	29.529	9.028	2.205	52.861	19.036
38	0.256	25.627	9.991	1.842	34.061	31.125
39				2.196	38.509	39.943
40				1.378	42.657	46.741
41				1.932	38.165	85.588
42				2.076	52.983	104.670
43				2.324	29.999	92.391
44				1.865	25.821	105.981
45				1.289	45.298	94.656
46				2.756	40.108	113.277
