# Supplementary material for: A Culex quinquefasciatus strain resistant to the binary toxin from Lysinibacillus sphaericus displays altered enzyme activities and energy reserves
Source: Parasit Vectors. 2023 Aug 9;16:273. doi: 10.1186/s13071-023-05893-z (PMC10413512; doi:10.1186/s13071-023-05893-z)
Supplement: Supplementary file 4 — Additional file 4: Table S4. Dataset of the protease activity assays with substrates for trypsins (Z-Phe-Arg-MCA), chymotrypsins (N-Ala-Ala-Phe-MCA) and aminopeptidases (l-Leu-MCA) in individual midguts of Culex quinquefasciatus early fourth instar larvae from a susceptible and a Bin-resistant strain. Protease activity (A; mU/midgut). Protein (P; µg/midgut). Specific activity (SA; U/g protein). [file 13071_2023_5893_MOESM4_ESM.pdf]

Additional file 4: Table S4. Dataset of the protease activity assays with substrates for trypsins (Z-Phe-Arg), chymotrypsins (Ala-Ala-Phe) and aminopeptidases (L-Leu) in individual midguts of *Culex quinquefasciatus* early fourth instar larvae from a susceptible and a Bin-resistant strain using different substrates. Protease activity (A; mU/midgut). Protein (P; µg/midgut). Specific Activity (SA; U/g protein).

Susceptible

N	Z-Phe-Arg			Ala-Ala-Phe			L-Leu		
	A	P	SA	A	P	SA	A	P	SA
1	0.002	4.980	0.322	0.212	21.586	9.822	0.260	17.743	14.670
2	0.001	2.543	0.481	0.160	19.622	8.133	0.767	36.262	21.160
3	0.018	19.524	0.921	0.727	53.950	13.473	0.118	6.915	17.100
4	0.066	35.016	1.887	0.295	29.842	9.888	0.484	27.450	17.617
5	0.016	13.776	1.127	0.557	47.970	11.609	0.249	13.764	18.101
6	0.023	33.949	0.685	0.198	32.433	6.095	0.511	44.380	11.504
7	0.032	23.411	1.363	0.078	19.539	3.993	0.369	21.266	17.337
8	0.005	8.349	0.578	0.043	9.046	4.786	0.154	23.295	6.630
9	0.002	13.205	0.184	0.337	18.040	18.653	0.518	49.578	10.451
10	0.044	25.114	1.764	0.401	38.620	10.371	0.556	36.378	15.296
11	0.051	34.666	1.461	0.204	32.870	6.219	0.459	30.527	15.035
12	0.055	32.871	1.661	0.619	42.315	14.628	0.595	26.100	22.789
13	0.008	10.414	0.759	0.387	30.466	12.687	0.206	12.720	16.233
14	0.033	22.839	1.425	0.492	34.969	14.077	0.239	14.784	16.160
15	0.049	19.693	2.484	0.373	30.835	12.092	0.119	9.904	12.062
16	0.012	29.529	0.398	0.055	15.855	3.450	0.054	18.829	2.879
17	0.001	10.063	0.133	0.017	4.798	3.464	0.035	10.838	3.206
18	0.001	8.892	0.122	0.033	13.119	2.537	0.072	19.188	3.745
19	0.006	15.837	0.361	0.066	22.541	2.922	0.039	17.237	2.254
20	0.009	21.931	0.427	0.064	30.358	2.121	0.083	18.646	4.451
21	0.010	32.221	0.297	0.050	18.798	2.654	0.071	22.707	3.113

22	0.006	22.544	0.274	0.063	14.274	4.392	0.066	20.082	3.293
23	0.000	3.352	0.078	0.048	20.917	2.311	0.064	21.201	3.030
24	0.006	17.207	0.355	0.054	24.297	2.240	0.081	23.447	3.452
25	0.008	17.781	0.459	0.019	8.441	2.279	0.019	6.547	2.827
26	0.001	10.410	0.122	0.058	15.708	3.671	0.019	14.757	1.316
27	0.007	27.043	0.260	0.074	30.612	2.421	0.066	14.534	4.525
28	0.006	26.280	0.383	0.086	42.388	4.352	0.055	19.149	9.955
29	0.004	26.654	0.485	0.031	16.234	4.296	0.150	28.528	6.925
30	0.001	10.250	0.363	0.027	18.764	5.411	0.060	18.063	3.795
31	0.010	42.716	0.314	0.129	34.887	4.089	0.047	16.659	6.607
32	0.010	29.061	0.302	0.107	37.145	3.163	0.122	25.958	4.463
33	0.004	20.100	0.386	0.055	35.739	2.811	0.102	19.131	5.969
34	0.008	19.712	0.681	0.198	45.552	4.381	0.098	27.978	5.824
35	0.014	28.602	0.520	0.159	36.945	2.700	0.084	24.118	4.855
36	0.010	28.182	0.343	0.222	40.949	5.543	0.128	33.502	4.932
37	0.008	25.778	0.566	0.126	30.928	4.368	0.108	37.452	5.888
38	0.008	28.019	0.611	0.082	25.865	3.225	0.028	8.026	6.122
39	0.007	17.069	0.582	0.037	13.036	4.726	0.106	27.061	6.771
40	0.014	20.132	0.243	0.112	25.600	2.034	0.226	22.751	2.877
41	0.018	34.991	0.151	0.079	29.100	1.880	0.131	18.954	5.264
42	0.006	16.264	0.106	0.199	35.896	1.417	0.107	28.295	3.296
43	0.010	17.421	0.238	0.159	36.315	3.702	0.176	26.704	2.839
44	0.025	41.092	0.361	0.064	19.781	2.879	0.105	23.459	4.691
45	0.021	36.862	0.180	0.128	27.157	1.541	0.219	36.700	5.310
46							0.193	33.142	3.486
47							0.146	30.090	3.468
48							0.160	32.342	3.822
49							0.085	14.505	2.889

N	Z-Phe-Arg			Ala-ala-Phe			L-Leu		
50							0.135	22.005	3.513
51							0.242	35.788	3.900
**Resistant**									
**N**	**Z-Phe-Arg**			**Ala-ala-Phe**			**L-Leu**		
	**A**	**P**	**SA**	**A**	**P**	**SA**	**A**	**P**	**SA**
1	0.062	36.220	1.700	0.081	6.718	12.059	0.162	8.969	18.079
2	0.096	52.495	1.824	0.286	21.601	13.233	0.087	6.251	13.913
3	0.028	23.944	1.187	0.194	19.329	10.058	0.238	12.748	18.651
4	0.028	22.021	1.252	0.335	28.711	11.651	0.216	13.270	16.310
5	0.048	35.195	1.355	0.193	16.846	11.433	0.168	14.654	11.452
6	0.011	22.649	0.505	0.106	18.307	5.772	0.174	12.749	13.652
7	0.006	10.406	0.539	0.421	38.997	10.791	0.213	18.505	11.496
8	0.009	11.694	0.803	0.083	10.543	7.849	0.288	18.104	15.903
9	0.007	9.048	0.728	0.114	19.103	5.990	0.180	13.398	13.404
10	0.019	23.641	0.798	0.145	13.440	10.757	0.263	16.291	16.174
11	0.056	37.553	1.499	0.411	21.632	18.999	0.429	32.671	13.117
12	0.036	30.949	1.173	0.048	11.182	4.269	0.220	16.913	13.009
13	0.047	30.977	1.510	0.531	29.701	17.868	0.214	16.326	13.104
14	0.018	26.621	0.659	0.243	14.124	17.222	0.184	15.156	12.158
15	0.017	17.476	0.976	0.156	10.730	14.584	0.276	23.259	11.867
16	0.005	29.078	0.170	0.018	5.816	3.141	0.026	14.953	1.762
17	0.022	30.389	0.711	0.058	21.102	2.751	0.031	14.303	2.191
18	0.010	29.501	0.336	0.077	21.986	3.502	0.055	37.425	1.468
19	0.026	44.967	0.575	0.030	11.045	2.683	0.028	34.467	0.811
20	0.013	39.622	0.318	0.030	17.686	1.720	0.068	41.902	1.613
21	0.007	25.964	0.277	0.106	39.419	2.681	0.043	36.088	1.180
22	0.006	20.103	0.323	0.019	14.699	1.297	0.048	51.255	0.941

23	0.011	28.614	0.385	0.084	53.846	1.562	0.050	34.618	1.431
24	0.012	27.427	0.445	0.064	40.056	1.606	0.064	29.210	2.194
25	0.005	14.788	0.345	0.047	28.734	1.621	0.018	25.850	0.697
26	0.021	31.858	0.663	0.051	43.710	1.160	0.015	37.317	0.400
27	0.013	49.949	0.252	0.049	37.686	1.311	0.054	15.124	3.551
28	0.002	12.157	0.169	0.050	20.267	2.459	0.053	14.129	3.751
29	0.006	14.953	0.416	0.036	10.972	3.305	0.053	29.549	1.802
30	0.004	11.240	0.316	0.123	32.646	3.777	0.054	15.732	3.418
31	0.004	15.803	0.260	0.106	17.949	5.884	0.095	18.961	5.007
32	0.005	18.750	0.276	0.070	10.741	6.497	0.105	29.704	3.531
33	0.022	43.437	0.504	0.106	17.700	5.972	0.076	15.642	4.847
34	0.010	21.826	0.452	0.188	28.348	6.615	0.109	17.331	6.271
35	0.005	18.541	0.259	0.274	39.653	6.906	0.079	14.283	5.565
36	0.007	20.778	0.334	0.107	25.051	4.260	0.135	18.455	7.337
37	0.004	18.005	0.238	0.118	23.551	5.006	0.103	19.342	5.321
38	0.005	19.712	0.274	0.034	10.413	3.312	0.095	21.779	4.356
39	0.023	38.934	0.589	0.052	15.476	3.367	0.081	20.970	3.866
40	0.007	19.798	0.343	0.029	16.404	1.741	0.147	27.303	5.386
41	0.002	9.965	0.168	0.016	15.094	1.077	0.070	13.351	5.215
42	0.002	12.235	0.132	0.208	43.418	4.799	0.123	24.112	5.096
43	0.004	18.498	0.219	0.140	33.250	4.215	0.080	15.990	4.973
44	0.002	10.537	0.199	0.051	14.410	3.574	0.132	18.752	7.018
45	0.003	19.311	0.131	0.090	34.403	2.605	0.149	34.901	4.267
46							0.095	28.102	3.395
47							0.203	40.664	5.003
48							0.080	19.549	4.080
49							0.102	22.836	4.447
50							0.118	21.792	5.424

51	0.062	17.094	3.636
