# Supplementary material for: A Culex quinquefasciatus strain resistant to the binary toxin from Lysinibacillus sphaericus displays altered enzyme activities and energy reserves
Source: Parasit Vectors. 2023 Aug 9;16:273. doi: 10.1186/s13071-023-05893-z (PMC10413512; doi:10.1186/s13071-023-05893-z)
Supplement: Supplementary file 5 — Additional file 5: Table S5. Dataset of assays of lipid quantification in pools of early fourth instar larvae (n = 20) and individual female samples of Culex quinquefasciatus from a susceptible and a Bin-resistant strain. [file 13071_2023_5893_MOESM5_ESM.pdf]

Additional file 5: Table S5. Dataset of assays of lipids quantification in pools of early fourth instar larvae (n= 20) and in individual female samples of *Culex quinquefasciatus* from a susceptible and a Bin-resistant strain.

**Larvae**				
	**Absorbance (525 nm)**		**Lipids (µg)**	
**N**	**Susceptible**	**Resistant**	**Susceptible**	**Resistant**
1	0.270	0.292	51.724	55.939
2	0.713	0.408	136.590	78.161
3	0.379	0.385	72.605	73.755
4	0.652	0.770	124.904	147.510
5	0.827	1.052	158.429	201.533
6	1.335	1.029	255.747	197.126
7	0.792	0.402	151.724	77.011
8	0.746	0.368	142.912	70.498
9	0.956	0.391	183.142	74.904
10	0.405	0.366	77.586	70.115
11	0.679	0.202	130.077	38.697
12	0.400	0.529	76.628	101.341
13	0.712	0.463	136.398	88.697
14	0.795	0.362	152.299	69.349
15	1.178	0.342	225.670	65.517
16	1.034	0.317	198.084	60.728
17	0.952	0.220	182.375	42.146
18	1.116	0.384	213.793	73.563
19	0.375	0.329	71.839	63.027
20	0.556	0.439	106.513	84.100
21	0.641	0.483	122.797	92.529
22	0.370	0.313	70.881	59.962
23	0.897	0.440	171.839	84.291
24	0.951	0.359	182.184	68.774
25	0.964	0.357	184.674	68.391
26	0.908	0.453	173.946	86.782
27	0.969	0.400	185.632	76.628
28	1.204	0.482	230.651	92.337
29	0.997	0.389	190.996	74.521
30	1.002	0.370	191.954	70.881
**Adults**				
	**Absorbance (525 nm)**		**Lipids (µg)**	
**N**	**Susceptible**	**Resistant**	**Susceptible**	**Resistant**
1	0.497	0.264	95.211	50.575
2	0.539	0.277	103.257	53.065
3	0.458	0.250	87.739	47.893
4	0.548	0.282	104.981	54.023
5	0.530	0.288	101.533	55.172
6	0.506	0.224	96.935	42.912
7	0.545	0.266	104.406	50.958

8	0.427	0.304	81.801	58.238
9	0.455	0.298	87.165	57.088
10	0.562	0.293	107.663	56.130
11	0.591	0.279	113.218	53.448
12	0.479	0.221	91.762	42.337
13	0.492	0.239	94.253	45.785
14	0.507	0.311	97.126	59.579
15	0.464	0.267	88.889	51.149
16	0.508	0.263	97.318	50.383
17	0.400	0.200	76.628	38.314
18	0.545	0.232	104.406	44.444
19	0.532	0.283	101.916	54.215
20	0.536	0.302	102.682	57.854
21	0.446	0.221	85.441	42.337
22	0.567	0.315	108.621	60.345
23	0.532	0.239	101.916	45.785
24	0.427	0.233	81.801	44.636
25	0.488	0.305	93.487	58.429
26	0.556	0.311	106.513	59.579
27	0.485	0.278	92.912	53.257
28	0.454	0.266	86.973	50.958
29	0.514	0.298	98.467	57.088
30	0.465	0.254	89.080	48.659
