# Supplementary material for: A Culex quinquefasciatus strain resistant to the binary toxin from Lysinibacillus sphaericus displays altered enzyme activities and energy reserves
Source: Parasit Vectors. 2023 Aug 9;16:273. doi: 10.1186/s13071-023-05893-z (PMC10413512; doi:10.1186/s13071-023-05893-z)
Supplement: Supplementary file 6 — Additional file 6: Table S6. Dataset of assays performed for the quantification of reducing sugars in pools of early fourth instar larvae (n = 40) and pools of female (n = 5) Culex quinquefasciatus from a susceptible and a Bin-resistant strain. Absorbance at 540 nm (Abs). Test sample incubated with amyloglucosidase (T). Control sample incubated without enzyme (C). Final reducing sugars amount (S). [file 13071_2023_5893_MOESM6_ESM.pdf]

Additional file 6: Table S6. Dataset of assays for the quantification of reducing sugars in pools of early fourth instar larvae (n=40) and in pools of females (n=5) of *Culex quinquefasciatus* from a susceptible and a Bin-resistant strain. Absorbance at 540 nm (Abs). Test sample incubated with amyloglucosidase (T). Negative control sample incubated without enzyme (C). Reducing sugar amount (S).

**Susceptible Larvae**						**Susceptible Adults**				
	**Abs (540 nm)**		**Glucose mg/ml**		**µg/pool**	**Abs (540 nm)**		**Glucose mg/ml**		**µg/pool**
**N**	**T**	**C**	**T**	**C**	**S**	**T**	**C**	**T**	**C**	**S**
1	0.737	0.538	0.553	0.373	252.9	0.582	0.426	0.413	0.271	198.3
2	0.651	0.506	0.475	0.344	184.2	0.516	0.409	0.353	0.256	136.0
3	0.619	0.412	0.446	0.258	263.0	0.403	0.382	0.250	0.231	26.7
4	0.718	0.504	0.536	0.342	271.9	0.306	0.128	0.162	0.001	226.2
5	0.836	0.667	0.643	0.490	214.8	0.440	0.337	0.284	0.190	130.9
6	0.714	0.564	0.532	0.396	190.6	0.421	0.297	0.267	0.154	157.6
7	0.744	0.549	0.560	0.383	247.8	0.515	0.388	0.352	0.237	161.4
8	0.721	0.580	0.539	0.411	179.2	0.444	0.347	0.287	0.199	123.3
9	0.725	0.488	0.542	0.327	301.2	0.527	0.388	0.363	0.237	176.7
10	0.387	0.206	0.236	0.071	230.0	0.414	0.307	0.260	0.163	136.0
11	0.712	0.577	0.531	0.408	171.5	0.356	0.299	0.208	0.156	72.4
12	0.600	0.415	0.429	0.261	235.1	0.484	0.290	0.324	0.148	246.6
13	0.665	0.508	0.488	0.345	199.5	0.464	0.203	0.306	0.069	331.7
14	0.678	0.433	0.500	0.277	311.3	0.524	0.339	0.360	0.192	235.1
15	0.769	0.540	0.582	0.375	291.0	0.406	0.349	0.253	0.201	72.4
	**Resistant Larvae**					**Resistant Adults**				
	**Abs (540 nm)**		**Glucose mg/ml**		**µg/pool**	**Abs (540 nm)**		**Glucose mg/ml**		**µg/pool**
**N**	**T**	**C**	**T**	**C**	**S**	**T**	**C**	**T**	**C**	**S**
1	1.081	0.800	0.866	0.611	0.255	0.566	0.341	0.398	0.194	0.204
2	0.909	0.764	0.710	0.578	0.132	0.587	0.366	0.417	0.217	0.201
3	1.048	0.710	0.836	0.529	0.307	0.593	0.340	0.423	0.193	0.230
4	1.099	0.703	0.882	0.523	0.359	0.599	0.320	0.428	0.175	0.253
5	1.088	0.925	0.872	0.724	0.148	0.533	0.347	0.368	0.199	0.169
6	1.333	0.978	1.094	0.772	0.322	0.526	0.397	0.362	0.245	0.117
7	0.874	0.548	0.678	0.382	0.296	0.409	0.291	0.256	0.149	0.107
8	0.867	0.750	0.671	0.565	0.106	0.532	0.312	0.367	0.168	0.200
9	1.220	0.720	0.992	0.538	0.454	0.444	0.291	0.287	0.149	0.139
10	1.270	0.872	1.037	0.676	0.361	0.549	0.375	0.383	0.225	0.158
11	1.197	0.962	0.971	0.758	0.213	0.590	0.398	0.420	0.246	0.174
12	1.162	0.873	0.939	0.677	0.262	0.577	0.335	0.408	0.188	0.220
13	1.206	1.059	0.979	0.846	0.133	0.440	0.249	0.284	0.110	0.173
14	0.958	0.757	0.754	0.572	0.182	0.510	0.365	0.347	0.216	0.132
15	1.351	1.093	1.111	0.877	0.234	0.501	0.338	0.339	0.191	0.148
