# Supplementary material for: A Culex quinquefasciatus strain resistant to the binary toxin from Lysinibacillus sphaericus displays altered enzyme activities and energy reserves
Source: Parasit Vectors. 2023 Aug 9;16:273. doi: 10.1186/s13071-023-05893-z (PMC10413512; doi:10.1186/s13071-023-05893-z)
Supplement: Supplementary file 7 — Additional file 7: Table S7. Dataset of assays to determine the fecundity and fertility of Culex quinquefasciatus females from a susceptible and a Bin-resistant strain under controlled or stressing rearing condition. Days post oviposition (dpo). First instar larvae percentage (LI%). Replicate (R). Standard deviation (SD). [file 13071_2023_5893_MOESM7_ESM.pdf]

Additional file 7: Table S7. Dataset of assays to determine the fecundity and fertility of *Culex quinquefasciatus* females from a susceptible and a Bin-resistant strain under controlled or stressing rearing condition. Days post oviposition (DPO). First instar larvae percentage (L1%). Replicate (R). Standard Deviation (SD).

**Controlled rearing condition-Susceptible**												
**DPO**		**R1**	**R2**	**R3**	**R4**	**R5**					**Mean**	**SD**
0	Egg N°	194	248	251	245						235	23.48
	L1%	97	95	95	98						96.3	1.30
3	Egg N°	247	229	238	279	268					252	18.63
	L1%	60	51	83	74	75					68.6	11.50
5	Egg N°	214	246	242	226	228					231	11.57
	L1%	41	52	57	59	60					53.8	6.97
8	Egg N°	276	238	223							246	22.31
	L1%	4	2	2							2.67	0.94
**Controlled rearing condition-Resistant**												
**DPO**		**R1**	**R2**	**R3**	**R4**	**R5**					**Mean**	**SD**
0	Egg N°	239	201	272	254						242	26.14
	L1%	90	95	91	94						92.5	2.06
3	Egg N°	242	224	238	279	268					250	20.24
	L1%	50	51	72	73	82					65.6	12.82
5	Egg N°	205	237	278	229	259					242	25.09
	L1%	50	43	61	51	57					52.4	6.18
8	Egg N°	215	243	262							240	19.30
	L1%	1	2	3							2	0.82
**Stress rearing condition-Susceptible**												
**DPO**		**R1**	**R2**	**R3**	**R4**	**R5**	**R6**	**R7**	**R8**	**R9**	**Mean**	**SD**
0	Egg N°	106	93	96	94	115	95				99.8	8.03
	L1%	90	88	90	91	91	89				89.8	1.07
3	Egg N°	98	118	113	126	115	112	124	34	99	104	26.47
	L1%	40	42	43	64	64	56	58	59	58	53.8	8.95
5	Egg N°	123	115	118	109	104	113	131	126	111	117	8.18
	L1%	30	27	30	28	31	30	35	33	32	30.7	2.31
8	Egg N°	119	106	112	107	114	118	106	127	109	113	6.74
	L1%	0	0	0	0	0	0	0	0	0	0	0.00
**Stress rearing condition-Resistant**												
**DPO**		**R1**	**R2**	**R3**	**R4**	**R5**	**R6**	**R7**	**R8**	**R9**	**Mean**	**SD**
0	Egg N°	142	137	104	98	110	98				115	17.97
	L1%	95	91	96	98	91	90				93.5	2.99
3	Egg N°	135	128	147	118	127	121	108	102	111	122	13.29
	L1%	60	55	60	72	73	64	70	69	69	65.8	5.92
5	Egg N°	132	141	136	132	126	119	109	121	103	124	11.85
	L1%	40	40	32	46	42	35	34	39	32	37.8	4.54
8	Egg N°	129	133	144	125	118	116	115	133	99	124	12.44
	L1%	0	0	0	0	0	0	0	0	0	0	0.00
